# Two Synthetic Approaches to Coinage Metal(I) Mesocates: Electrochemical versus Chemical Synthesis

**DOI:** 10.1021/acs.inorgchem.2c02243

**Published:** 2022-08-19

**Authors:** Sandra Fernández-Fariña, Miguel Martínez-Calvo, Marcelino Maneiro, José M. Seco, Guillermo Zaragoza, Ana M. González-Noya, Rosa Pedrido

**Affiliations:** † Departamento de Química Inorgánica, Facultade de Química, Campus Vida, Universidade de Santiago de Compostela, 15782 Santiago de Compostela, Spain; ‡ Departamento de Química Inorgánica, Facultade de Ciencias, Campus Terra, Universidade de Santiago de Compostela, 27002 Lugo, Spain; § Departamento de Química Orgánica, Facultade de Química, Campus Vida, Universidade de Santiago de Compostela, 15782 Santiago de Compostela, Spain; ∥ Unidade de Difracción de Raios X, Edificio CACTUS, Campus Vida, Universidade de Santiago de Compostela, 15782 Santiago de Compostela, Spain

## Abstract

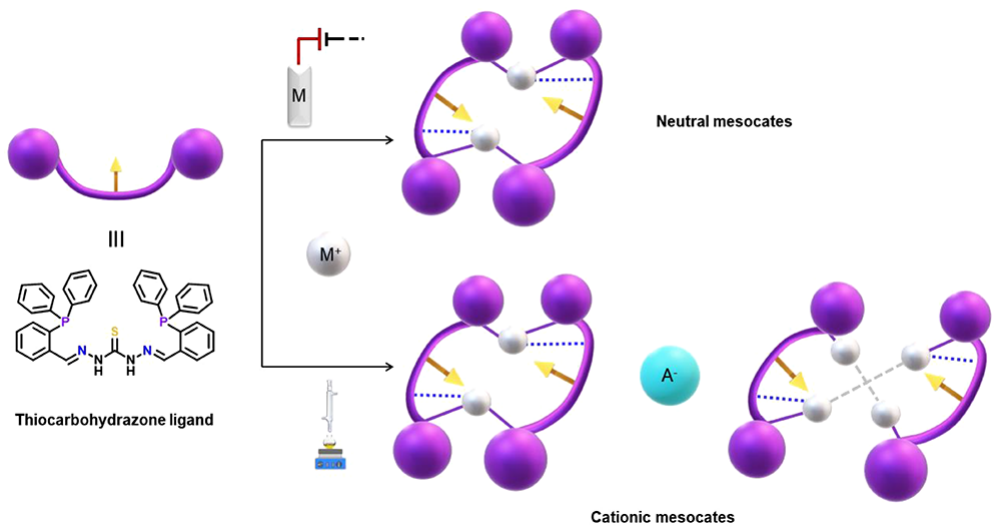

We report two different approaches to isolate neutral and cationic mesocate-type metallosupramolecular architectures derived from coinage monovalent ions. For this purpose, we use a thiocarbohydrazone ligand, H_2_L (**1**), conveniently tuned with bulky phosphine groups to stabilize the M^I^ ions and prevent ligand crossing to achieve the selective formation of mesocates. The neutral complexes [Cu_2_(HL)_2_] (**2**), [Ag_2_(HL)_2_] (**3**), and [Au_2_(HL)_2_] (**4**) were prepared by an electrochemical method, while the cationic complexes [Cu_2_(H_2_L)_2_](PF_6_)_2_ (**5**), [Cu_2_(H_2_L)_2_](BF_4_)_2_ (**6**), [Ag_2_(H_2_L)_2_](PF_6_)_2_ (**7**), [Ag_4_(HL)_2_](NO_3_)_2_ (**8**), and [Au_2_(H_2_L)_2_]Cl_2_ (**9**) were obtained by using a metal salt as the precursor. All of the complexes are neutral or cationic dinuclear mesocates, except the silver nitrate derivative, which exhibits a tetranuclear cluster mesocate architecture. The crystal structures of the neutral and cationic copper(I), silver(I), and gold(I) complexes allow us to analyze the influence of synthetic methodology or the counterion role on both the micro- and macrostructures of the mesocates.

## Abstract 2

Two synthetic approaches applied to a thiocarbohydrazone ligand lead to neutral or cationic mesocate-type metallosupramolecular architectures with coinage monovalent ions.

## Introduction

In the recent years, a wide variety of metallosupramolecular architectures obtained through self-assembly processes between organic ligands and metal ions have been published, among which helicates and mesocates can be highlighted.^1−7^

Mesocates or helicates are composed of at least two organic strands and two metal ions. If the ligands adopt a twisted arrangement around the metal ions, homochiral racemic helicates are formed, whereas if the ligands coordinate to metal ions without crossing each other,^8^ achiral mesocates are obtained. In a same manner, mesocates are complexes that could be seen as the midpoint of two helicates of opposite hand.

To date, supramolecular research has mainly focused on helicates because their simplicity has facilitated the study of inherent factors directing the self-assembly process and their similarity to the DNA double helix has made new approaches to potential metallodrugs possible.^9−12^ In spite of this, mesocates must be considered as fascinating as helicates because they also exhibit high potential in different fields such as magnetism,^13−17^ luminescent molecular sensors,^18−20^ or pharmacology,^21−23^ among others. For that reason, the development of synthetic routes to obtain mesocates on selective processes is of great interest nowadays and deserves to be investigated.

Since Albrecht and Kotila reported the first mesocate case^8^ and established the well-known “odd–even rule” referring to the length of the spacer in the ligand, we and other authors have highlighted the difficulty in controlling the factors that allow the selective formation of mesocates or helicates.^24^ In this context, different aspects such as the ligand design,^8,25,26^ the nature of the metal ion,^24,27^ the experimental conditions (solvent, temperature, etc.),^24^ or the inclusion of guest molecules^20^ have been investigated. All of these studies have mainly been focused on the ligand design and/or divalent metal ions.^8,25,27^ In contrast, studies with M^I^ coinage metal ions are scarce, and no routes for the isolation of metal(I) mesocates/helicates have been reported. Thus, only a few examples of copper(I),^18,26,28^ silver(I),^29−31^ and gold(I)^22^ mesocates have been published to date.

Thiosemicarbazone ligands can be considered as one of the most versatile kernels in chemistry.^32^ Through decades of intensive research, thousands of different thiosemicarbazone compounds have been achieved. The exhaustive research performed in thiosemicarbazones may be attributed to their versatility on coordination, their utility to form diverse heterocycles, and their proven biological activities, like antitumor, metastatic, and antibacterial, among many others.^33^ Over the past few years, an increased interest in thiocarbohydrazone ligands, which may be considered as extended thiosemicarbazones, has emerged.^34^ Thus, thiocarbohydrazones possess two more donor atoms in their skeleton compared to thiosemicarbazones and therefore could potentially form a wider variety of metallosupramolecular structures. Surprisingly, a reduced number of thiocarbohydrazone metal complexes has been reported to date.^34^ Among them, grids^35,36^ and mononuclear^37^ and dinuclear^38^ species have been described. Also, examples of silver(I) clusters derived from these types of ligands have been found.^39,40^ In addition, no examples of donor NSP-thiocarbohydrazone ligands have been found in the literature.

Our research group has pioneered the application of an electrochemical procedure for the isolation of neutral metal complexes with singular supramolecular arrangements. This methodology, combined with the use of thiosemicarbazones as ligands, allowed us to isolate cluster helicates with monovalent metal ions, whereas lineal helicates or mesocates were assembled with divalent metal ions.^24,27,41−44^

**Scheme 1:**
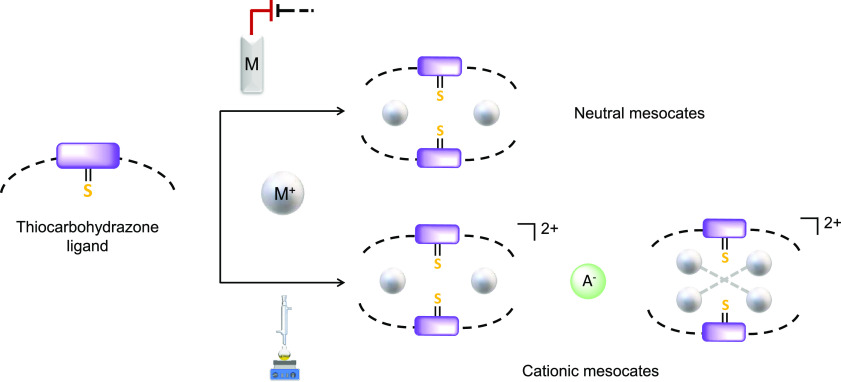
Selective Isolation of Mesocates Presented in This Work

Taking all of these considerations in mind, herein we report a double and efficient route focused on the preparation of neutral and ionic mesocates with monovalent coinage metal ions (Scheme 1).

**Figure 1:**
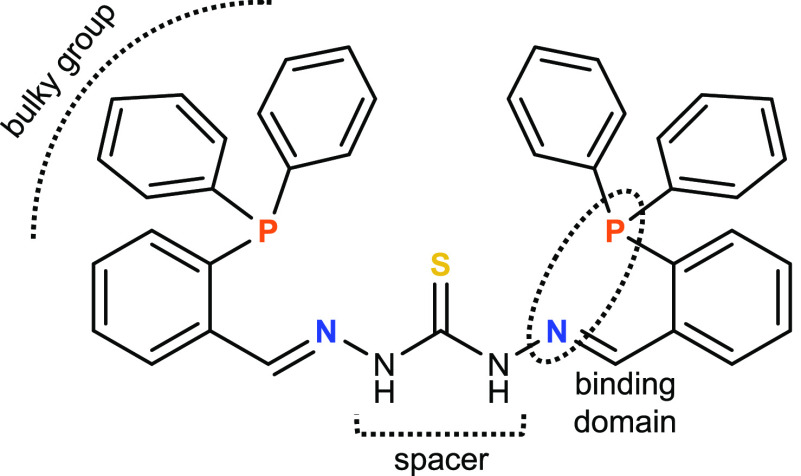
Representation of ligand **1**.

Our strategy is based on a conveniently functionalized thiocarbohydrazone strand. In this sense, we have designed the thiocarbohydrazone ligand H_2_L (**1**), which incorporates two phosphine groups (Figure 1) that fit with the proposed requirements for the isolation of metal(I) mesocates. In this sense, ligand **1** is equipped with (i) two bulky triphenylphosphine binding domains that each contain a phosphorus soft donor atom to stabilize M^I^ coinage ions and hinder crossing of the organic strands and (ii) a spacer containing a soft donor atom that may coordinate to the metal ions and at the same time prevent ligand crossing to favor the selective formation of mesocates.

## Results and Discussion

**Scheme 2:**
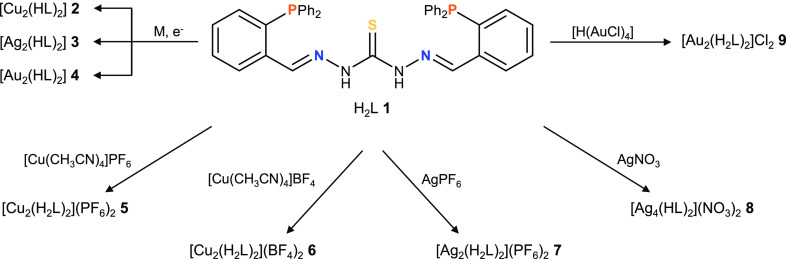
Complexes **2**–**9** Synthesized in This Work

In this work, we have carried out the synthesis of both neutral and cationic copper(I), silver(I), and gold(I) mesocates (Scheme 2) derived from the thiocarbohydrazone ligand **1** by using two different methodologies. Neutral complexes were obtained using an electrochemical procedure (**2**–**4**), whereas cationic mesocates (**5**–**9**) were isolated from different metallic salts like [Cu(CH_3_CN)_4_]PF_6_, [Cu(CH_3_CN)_4_]BF_4_, AgPF_6_, AgNO_3_, and H[AuCl_4_] (see the Experimental Section). The main objective was to study the influence of different factors such as the synthetic procedure, the counterion, and the M^I^ ion size on the final stoichiometry and/or architecture of the complexes.

### Thiocarbohydrazone Ligand H_2_L

The phosphinethiocarbohydrazone ligand **1** (Figure 1) can be described as bicompartmental and potentially pentadentate [N_2_SP_2_]. **1** was obtained by the reaction of 2-diphenylphosphinobenzaldehyde and thiocarbohydrazide in a 2:1 ratio and fully characterized using a wide variety of techniques, as detailed in the Experimental Section and shown in Figures S1–S6.

### Self-Assembly of Neutral Mesocates by Electrochemical Synthesis

The electrochemical methodology is a simple, efficient, and inexpensive technique that allows metallosupramolecular architectures to be obtained directly from redox processes that involve the oxidation of a free metal plate and the reduction of the precursor organic ligand (section 1). In addition, it is carried out at room temperature with pure reagents instead of metal salts, avoiding in many cases a possible competition between the anion and ligand during coordination to the metal ion.^45,46^

Electrochemical monooxidation of a metal plate (copper, silver, and gold) in a conducting acetonitrile (CH_3_CN) solution of ligand **1** afforded orange (copper and silver) or yellow (gold) solids, which were readily characterized. The analytical data and mass spectrometry (MS) spectra (Figure S7) for these solids are consistent with the formation of the neutral metal(I) dimeric complexes [M_2_(HL)_2_] (**2**–**4**), which arise from the monodeprotonation of ligands in solution. These formulations agree with the molar conductivity values of 2–10 μS·cm^–1^, typical for nonelectrolyte compounds [10^–3^ M *N*,*N*-dimethylformamide (DMF) solutions].^47^ The solids were also characterized by IR, ^1^H and ^31^P NMR, and UV–vis spectroscopy studies (Figures S8–S10 and Table S1).

The three neutral complexes share some similar features in the IR spectra. Thus, coordination of the ligand to the metal ions leads to a displacement on the vibrational bands to larger wavelengths compared to the free ligand (Figure S8), indicating coordination of the ligand through the imine nitrogen and sulfur atoms.

To study the properties of the neutral complexes in solution, we performed ^1^H and ^31^P NMR experiments at room temperature using DMSO-*d*_6_ as the solvent. The ^1^H NMR spectra of these compounds generally show a displacement of the signals to low field with respect to the free ligand and a broadening of the aromatic signals (Figure S9). This effect can be attributed to coordination of the ligand to the M^I^ metal centers (M = Cu, Ag, and Au). The ^31^P NMR spectra of the complexes exhibit a displacement of the signals to low field with respect to the free ligand, which confirms coordination of the phosphorus atom to the metal ions (Figure S10). It should be highlighted that in the case of the silver(I) complex **3**, a doublet appears at 6.19 ppm because of the ^107^Ag–^31^P coupling. The value of the coupling constant in this case (*J* = 364.1 Hz) indicates that two phosphorus atoms are coordinated to each metal ion.^48^ Also, in gold(I) complex **4**, a single singlet appears at 34.57 ppm, showing that in solution the four phosphorus atoms are equivalent.

### X-ray Structures of Neutral Mesocates

**Figure 2:**
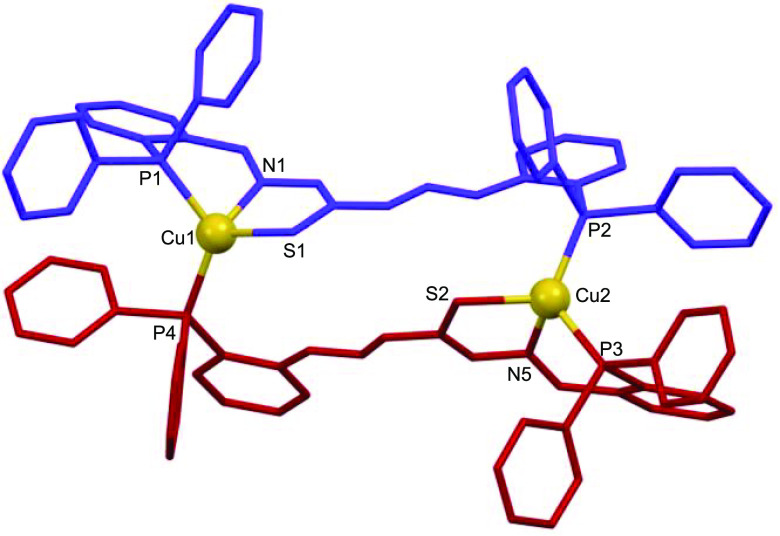
Crystal structure of the copper(I) mesocate 2*.

**Figure 3:**
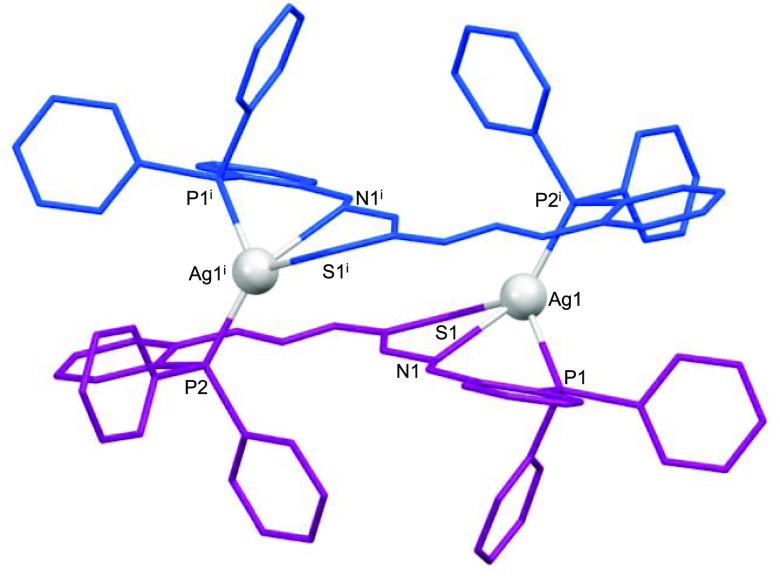
Crystal structure of the silver(I) mesocate 3*.

**Figure 4:**
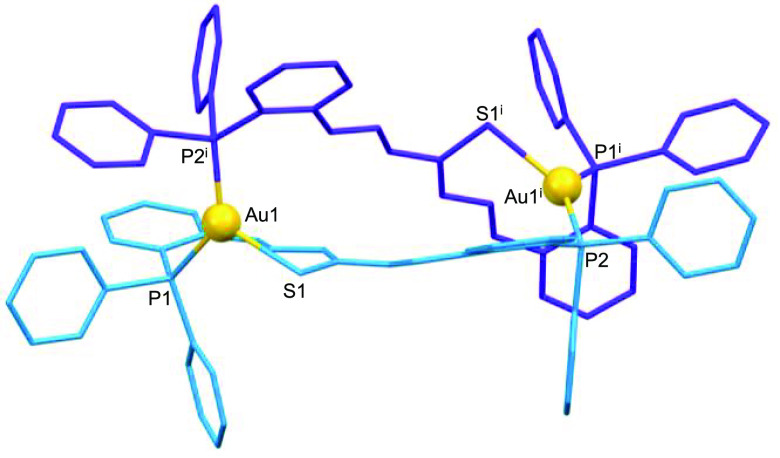
Crystal structure of the gold(I) mesocate 4*.

Slow evaporation of the mother liquors from the syntheses of **2** and **3** or recrystallization in chloroform/hexane of solid **4** allowed us to obtain orange crystals suitable for X-ray diffraction studies. The structures revealed the formation of neutral dinuclear mesocates of [Cu_2_(HL)_2_]·3.5CH_3_CN (2*; Figure 2), [Ag_2_(HL)_2_]·4CH_3_CN (3*; Figure 3), and [Au_2_(HL)_2_]·8CHCl_3_ (4*; Figure 4) stoichiometries. Selected crystal data are collected in the Supporting Information. Relevant bond lengths and angles for these structures are compiled in Tables S2–S4.

Every mesocate unit is composed of two monoanionic bridging ligands [HL]^−^. In the case of the copper and silver mesocates, the metal ions are coordinated to the imine nitrogen, phosphorus, and central thioamide sulfur atoms of one ligand strand, completing tetracoordination with the phosphorus atom of the second ligand unit, thus generating a [P_2_NS] tetrahedral distorted environment.

In the gold mesocate 4*, each gold ion is bound to the phosphorus and central thioamide sulfur atoms of one ligand strand and the phosphorus atom of the second ligand unit, exhibiting a [P_2_S] trigonal-planar distorted kernel. However, a weak interaction with the imine nitrogen atom (Au1–N1, 2.65 Å) cannot be ruled out.^49^

These coordination modes result in 18-membered metallomacrocyclic rings for each complex with dimensions of ca. 10.2 × 3.2 Å for copper, 9.1 × 3.1 Å for silver, and 10.0 × 3.6 Å for gold complexes (Figures S11–S13). The intradinuclear M–M distances are 8.475, 6.312, and 9.047 Å for copper, silver, and gold complexes, respectively, which precludes metal–metal interactions.

**Figure 5:**
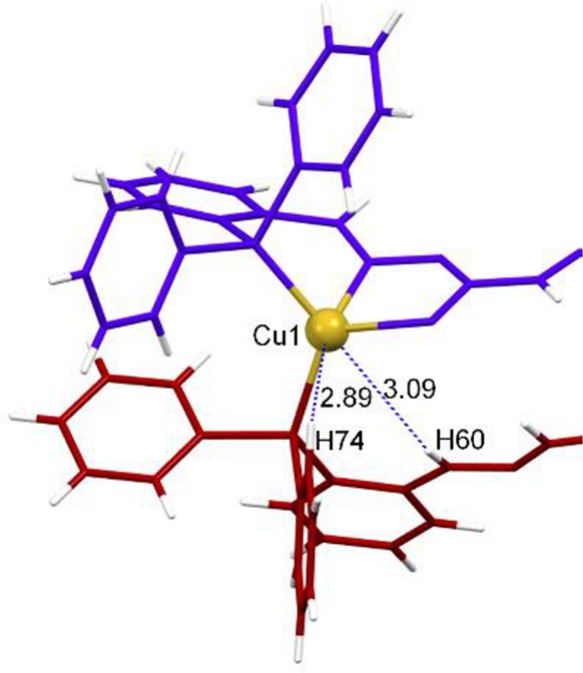
Intramolecular agostic interactions (CH60···Cu1 3.02 Å, CH74···Cu1 2.89 Å, CH21···Cu2 2.89 Å, and CH33···Cu2 3.01 Å) in 2*.

The assembly of neutral mesocates confirms that the two bulky phosphine groups in ligand **1** avoid crossing of the ligand threads and ensures the formation of mesocates instead of helicates. Moreover, the mesocate arrangement of the ligand maximizes the number of weak intramolecular interactions of the types of CH−π, hydrogen-bonding, and agostic contacts (Figures 5 and S14–S16),^49,50^ thus contributing to the mesocate assembly process.

In addition, the ligand incorporates the central sulfur donor atom in one of the PN binding domains, acting as PNS/PN for one of the metal ions and monodentate P for the second one. The behavior of the sulfur atom as a monodentate donor also favors the mesohelical arrangement because a M–S–M bridging behavior would lead to the formation of cluster metal(I) complexes, as was found before.^40,51^

On the other side, the electrochemical synthetic procedure plays a key role in mesocates formation because it allows precise control of the electrochemical conditions to achieve monodeprotonation of the ligand. This control refers to the reaction time that relates to the metal oxidation state and deprotonation degree in the ligand. In the herein-exposed case, bideprotonation of the ligand would presumably result in tetranuclear copper(I) cage assembly or oxidation to copper(II) complexes, as reported by Dragancea and coauthors.^52^ In order to confirm this prediction, we performed electrochemical synthesis of the complexes in bideprotonation conditions. The compounds isolated correspond to M_4_L_2_ species, as indicated by elemental analysis, IR spectroscopy, and MS (the spectra of Cu_4_L_2_ are shown as examples in Figures S17 and S18).

### Self-Assembly of the Cationic Mesocates

Complexes **5**–**9** were synthesized by the reaction of **1** with the corresponding metallic salt (in 1:1 ratio): **5** from [Cu(CH_3_CN)_4_]PF_6_, **6** from [Cu(CH_3_CN)_4_]BF_4_, **7** from AgFP_6_, **8** from AgNO_3_, and **9** from reduced [H(AuCl_4_)]. They were readily characterized by different techniques (see the Experimental Section and Figures S19–S27). Characterization data and MS spectra allowed us to propose dicationic dinuclear species [M_2_(H_2_L)_2_]^2+^ involving the neutral ligand in the case of compounds **5**–**7** and **9** and a dicationic tetranuclear stoichiometry [Ag_4_(HL)_2_]^2+^ for compound **8**, being in this case the ligand acting in a monoanionic mode. These formulations are in agreement with the measured molar conductivity values, in the range of 133–156 mS cm^–1^, typical for 1:2 electrolyte compounds (10^–3^ M DMF solutions).^47^ The solids were also characterized by IR, ^1^H NMR, and UV–vis, obtaining a pattern similar to that found in neutral mesocates. It should be highlighted that, in the IR spectra of complexes **5**–**8**, the characteristic bands corresponding to the counterions (PF_6_^–^, BF_4_^–^, and NO_3_^–^) can be clearly identified (Figures S19–S22). It is also remarkable that in the MS spectra of the silver complexes a fragment containing the counterion {Ag_2_(H_2_L)_2_]PF_6_}^+^ can be observed for **7** (Figure S23) and the tetranuclear signal [Ag_4_(HL)_2_-H]^+^ for the cluster **8** (Figure S24).

### X-ray Structures

Slow evaporation of the mother liquors resulting from the synthesis of compounds **5**–**9** allowed us to obtain suitable crystals from which the molecular structure was determined by X-ray crystallography. The main crystal data are collected in the Supporting Information. Selected bond lengths and angles for these structures are collected in Tables S5–S9. In all cases, the bond distances M–S, M–N, and M–P (M = Cu^I^, Ag^I^, and Au^I^) are in the range expected for complexes derived from thiocarbohydrazone ligands.^37,39^

The crystal structures revealed that [Cu_2_(H_2_L)_2_](PF_6_)_2_·CH_3_CN·2H_2_O (5*), [Cu_2_(H_2_L)_2_](BF_4_)_2_·5CH_3_CN (6*), [Ag_2_(H_2_L)_2_](PF_6_)_2_·6CH_3_CN (7*), and [Au_2_(H_2_L)_2_]Cl_2_·6.2CH_3_OH (9*) consist of discrete dicationic dinuclear mesocates [M_2_(H_2_L)_2_]^2+^ structurally similar to those obtained by electrochemical synthesis (2*–4*). In the case of the silver complex prepared with silver nitrate, a dicationic tetranuclear silver cluster mesocate [Ag_4_(HL)_2_](NO_3_)_2_·4CH_3_OH (8*) was isolated, thus confirming the singular stoichiometry found for the solid **8**.

Analyzing the structures by metal ion, the copper complexes (5* and 6*; Figures S27 and S28) give rise to mesohelical architectures similar to those isolated by electrochemical synthesis (Figure 2), where copper atoms are coordinated to a sulfur atom, an imine nitrogen atom, and a phosphorus atom of one ligand and a phosphorus atom of another ligand site, giving a [P_2_NS] tetrahedral distorted environment (Tables S5 and S6).

**Figure 6:**
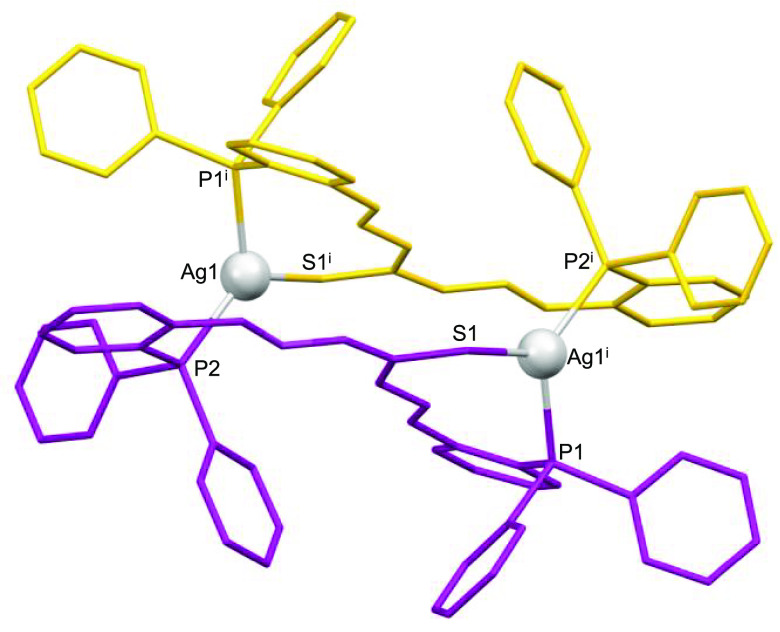
Crystal structure of the dicationic silver(I) mesocate 7*.

**Figure 7:**
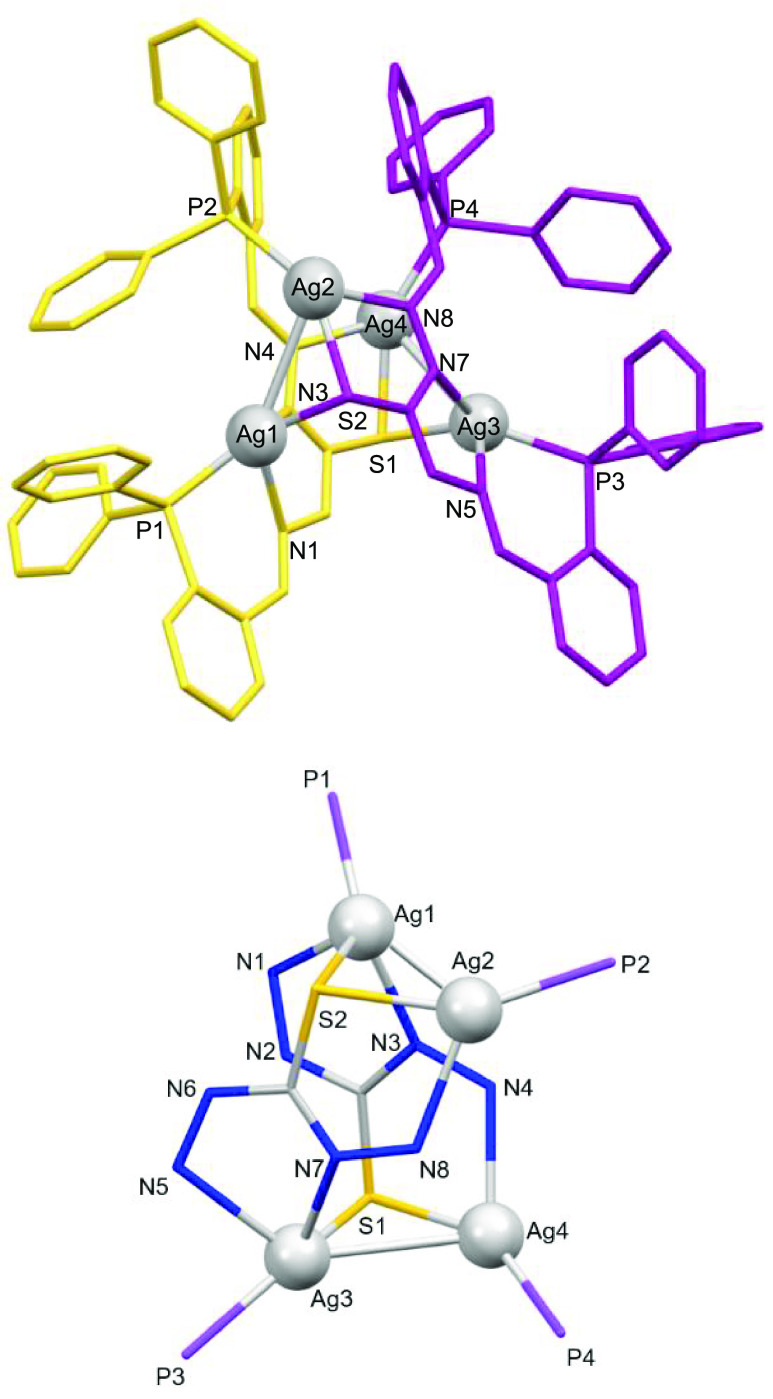
Crystal structure of the tetranuclear silver(I) cluster 8* (above). Counterions and hydrogen atoms have been omitted for clarity. Cluster core representation in 8* (below).

**Figure 8:**
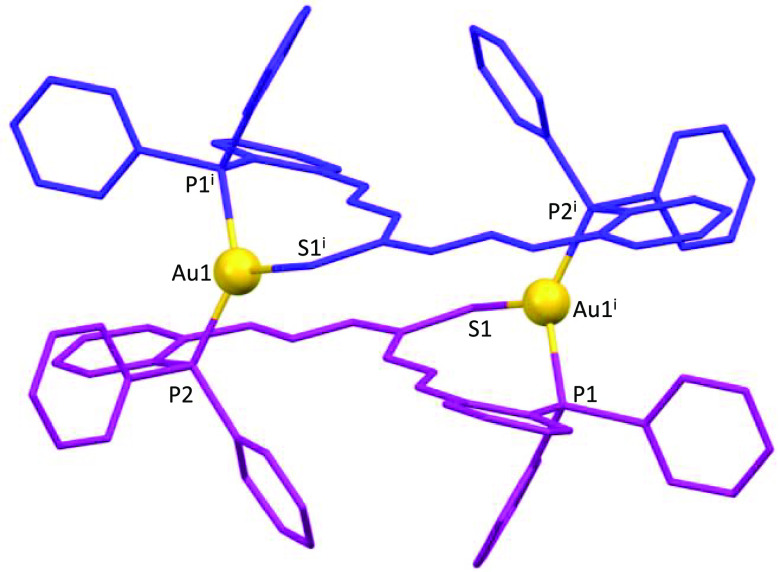
Crystal structure of the dicationic gold(I) mesocate 9*. Counterions and hydrogen atoms have been omitted for clarity.

In the case of the cationic silver complexes, we have obtained two different architectures: 7* (Figure 6) and 8* (Figure 7). Compound 7* (Figure 6) exhibits two silver atoms coordinated to the thioamide sulfur atom and the phosphorus atom of one ligand site and the phosphorus atom of another ligand site, giving a [P_2_S] distorted trigonal-planar environment (Table S7). We must highlight that the coordination mode is different compared to the neutral silver mesocate obtained by an electrochemical procedure, where we have observed a [P_2_NS] distorted tetrahedral environment (Figure 3). Therefore, we can conclude that the methodology does not affect the global mesohelical structure but does affect the microstructure of both silver(I) mesocates.

Nevertheless, the structure of the silver nitrate compound 8* (Figure 7) is a dicationic tetranuclear silver complex where the ligands are coordinated in its monoanionic form [HL]^−^ to silver metal ions without crossing each other. Also, 8* exhibits Ag–Ag bonds, giving rise to a cluster mesocate structure.

In this structure, Ag1 and Ag3 atoms are bound to the imine and thioamide nitrogen atoms, to the phosphorus atom of one ligand thread, and to the sulfur atom of the second ligand site. Furthermore, each metal ion is bound to another metal ion, assuming a [PN_2_SAg] distorted square-pyramidal environment, whereas metal ions Ag2 and Ag4 assume a [PNSAg] tetrahedral distorted environment (Table S8).

Each sulfur atom acts as μ_2_-S–Ag. The distance between the pairs Ag1–Ag2 (3.325 Å) and Ag3–Ag4 (3.140 Å), although larger than the metallic silver bond (2.889 Å),^53^ is lower than the sum of the van der Waals radii for both silver atoms (3.44 Å).^54^ Thus, the existence of argentophilic interactions can be considered.^55,56^ We must remark herein that two named cluster mesocates, M_2_(L^4^)_3_I_2_ (M = Cu^I^ and Au^I^), were published before.^28^ However, after careful analysis of these examples, they did not show metal–metal interactions (M–M distances of 12.00–12.12 Å). For that reason, to the best of our knowledge, this is the first example of a real cluster mesocate with coinage metal ions.

Bond distances Ag–S are in the expected range, giving rise to an asymmetric bridge. Bond distances Ag–N are also in the expected range, being larger than those found in the literature.^24^ The phosphorus atoms are oriented, avoiding unfavorable steric interactions (Figure 7).

Analysis of the two silver(I) structures obtained from silver(I) salts demonstrates that the ability of the counterion to deprotonate the ligand determines the resulting nuclearity of the cationic silver mesocates: a dinuclear mesocate was obtained in the case of the PF_6_^–^ salt, whereas a tetranuclear mesocate was assembled when the counterion was NO_3_^–^. In addition, in the case of dinuclear silver mesocates (neutral 3* and cationic 7*), we can establish that the methodology affects the microstructure because we observe two different coordination environments in these two complexes. In addition, the presence/absence of a counterion depending on the synthetic methodology employed is also relevant for the final nuclearity of the mesocate. Thus, we have observed that, although the ligand acts as monodeprotonated in the neutral silver complex 3* and the cationic silver complex 8*, 3* shows a dinuclear architecture, whereas 8* derived from nitrate salt presents a tetranuclear cluster structure.

In the case of gold, the chemical synthesis was performed with a chloride precursor, giving rise to the crystalline dinuclear mesocate 9* (Figure 8), where each metal ion is coordinated to a thioamide sulfur and one phosphorus atom of the ligand site and a phosphorus atom of another ligand, giving a [P_2_S] trigonal-planar distorted environment (Table S9).

Furthermore, similar to neutral mesocates (2*–4*), weak agostic interactions are established between the C–H protons of the phenyl phosphines and the M^I^ ions (CH35···Cu1 2.72 Å in complex 5*; CH29···Cu1 2.86 Å in complex 6*; CH29···Ag1 2.99 Å in complex 7*; CH35···Au1 3.00 Å in complex 9*).

**Figure 9:**
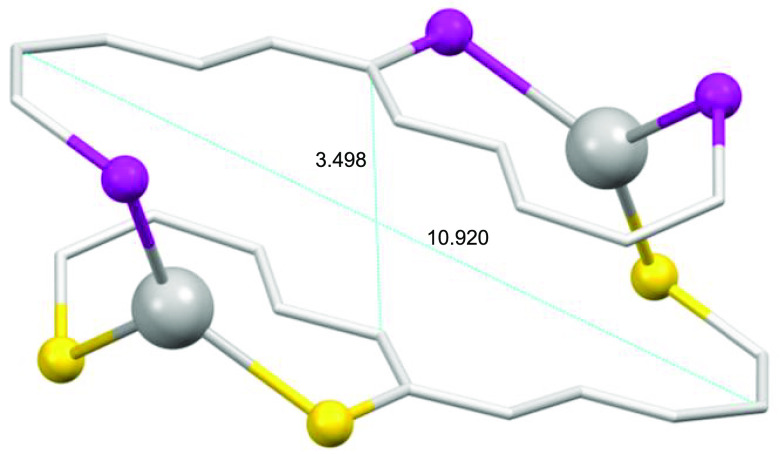
Metallomacrocycle ring featured in 7*.

Similar to those mesocates obtained by electrochemical synthesis (2*–4*), phosphine groups exhibit an anti conformation to avoid unfavorable steric interactions, resulting in the formation of 20-membered metallomacrocycles in the case of copper(I) mesocates (5* and 6*), while in the case of the silver(I) mesocate 7* (Figure 9) and gold(I) mesocate 9*, 28-membered metallomacrocycles were formed.

The distance between metal ions is large for all ionic dinuclear mesocates [Cu1–Cu2 6.093 Å (5*); Cu1–Cu1^i^ 6.389 Å (6*); Ag1–Ag1^i^ 6.566 Å (7*); Au1–Au1^i^ 6.704 Å (9*)] so that metallophilic interactions are excluded (∼2.8–3.4 Å).^54^ These distances are significantly smaller than those found in neutral mesocates for the copper(I) and gold(I) derivatives and on the same order as the case of the silver(I) complex.

## Conclusions

In this work, we have presented a feasible synthetic double approach to mesocates using a diphosphinethiocarbohydrazone ligand. The introduction of two phosphorus atoms in the ligand donor set ensures stabilization of the M^I^ coinage metal ions. In parallel, the presence in the ligand of the two bulky phosphines avoids crossing of the ligand threads, giving rise to the assembly of mesocates instead of helicates.

The electrochemical methodology allowed us to obtain dinuclear neutral mesocates, [M_2_(HL)_2_], whereas cationic dinuclear mesocates [M_2_(H_2_L)_2_]X_2_ were obtained if we used coinage salts. In the case of silver(I), two different structures were isolated, a tetranuclear mesocate, [Ag_4_(HL)_2_]X_2_, with a NO_3_^–^ counterion and a dinuclear mesocate, [Ag_2_(H_2_L)_2_]X_2_, with X = PF_6_^–^, thus demonstrating that the counterion influences the nuclearity of the cationic mesocates.

Analysis of the crystal structures shows that it is possible to isolate mesocate species independently of the monovalent metal ion used.

Overall, the reported results demonstrated once again the importance of the ligand design in the selective obtainment of metallosupramolecular architectures.

## Experimental Section

### Materials and Methods

Thiocarbohydrazide, 2-diphenylphosphinobenzaldehyde, tetrakis(acetonitrile)copper(I) hexafluorophosphate, tetrakis(acetonitrile)copper(I) tetrafluoroborate, silver hexafluorophosphate, silver nitrate, tetrachloroauric(III) acid salts, copper, silver, and gold plates, and all solvents were purchased from commercial sources and used without any purification. Melting points were determined using a Buchi 560 instrument. Elemental analysis of the compounds (C, H, N, and S) was performed with a Fisons EA model 1108 analyzer. Positive-ion electrospray ionization (ESI^+^) MS data were registered using a Bruker Microtof mass spectrometer. A Varian Mercury 300 spectrometer was employed to record the ^1^H NMR spectra operating at room temperature using DMSO-*d*_6_ or CD_3_CN as the deuterated solvent. Variable-temperature ^1^H NMR experiments in deuterated acetone and ^13^C and ^31^P NMR were performed on an Bruker Agilent AVIII-500. Chemical shifts are reported as δ (ppm). IR spectra were recorded from 400 to 4000 cm^–1^ on a Bruker FT-MIR VERTEX 70 V spectrophotometer using KBr pellets. A Crison micro CM 2200 conductivity meter was used to measure the conductivity values from 10^–3^ M solutions in DMF at room temperature. UV–vis absorption spectra were recorded from solutions of ca. 10^–5^ M in acetonitrile at room temperature using a Jasco UV–vis spectrophotometer.

### Thiocarbohydrazone Ligand H_2_L (**1**)

A total of 0.93 g (3.2 mmol) of 2-diphenylphosphinobenzaldehyde and 0.17 g (1.6 mmol) of thiocarbohydrazide were mixed and dissolved in absolute ethanol (50 mL). Then a catalytic amount of *p*-toluensulfonic acid was added to promote iminic condensation. The reaction mixture was refluxed for 3 h using a Dean–Stark trap to remove the released water. The resulting solution was cooled to 4 °C until the formation of a yellow product was observed. This solid was filtered off and washed with diethyl ether. Yield: 0.96 g (92%). Mp: 195–200 °C. Elem anal. Found: C, 71.2; H, 5.0; N, 8.5; S, 4.6. Calcd for C_39_H_32_N_4_P_2_S: C, 71.9; H, 5.0; N, 8.6; S, 4.9. ESI^+^ MS: *m*/*z* 651.2 ([H_2_L + H]^+^), 667.2 ([H_2_L(O) + H]). ^1^H NMR (500 MHz, acetone-*d*_6_, 278 K): δ 11.52 (s, 1H, H_1_), 11.01 (s, 1H, H_1_), 9.21 (d, *J* = 3.5 Hz, 1H, H_2_), 8.65 (d, *J* = 3.5 Hz, 1H, H_2_), 8.26 (m, 1H, H_3_), 7.95 (m, 1H, H_3_), 7.22 (t, *J* = 7.0 Hz, 4H, H_4_ + H_5_), 7.49 (m, 14H, H_Ar_), 7.18 (t, *J* = 7.0 Hz, 4H, H_Ar_), 6.85 (m, 2H, H_6_). ^13^C/DEPT NMR (500 MHz, DMSO-*d*_6_): δ 175.35 (C=S), 136.11 (C=N), 136.04 (C=N), 133.98–129.34 (CH_Ar_). ^31^P NMR (500 MHz, DMSO-*d*_6_): δ −10.15 (P^III^), −14.69 (P^III^). IR (KBr, cm^–1^): ν(N–H) 3138, ν(C–H) 2915, ν(C=N + C–N) 1585, 1522, 1477, 1432, ν(C=S) 1142, 745, 686. UV–vis (λ_max_, nm): 276, 332.

### Neutral Metal Complexes

The copper(I), silver(I), and gold(I) neutral complexes were prepared by using an electrochemical procedure. The electrochemical cell can be summarized as . As an example, the synthetic procedure used for the isolated complex [Ag_2_(HL)_2_] **3** is as follows:

A total of 0.1 g of **1** (0.154 mmol) was dissolved in acetonitrile (75 mL), and a small amount of tetraethylammonium perchlorate was added to the media as a supporting electrolyte. The resulting solution was electrolyzed at 5 mA and 5 V at room temperature for 50 min, and the orange solid obtained was isolated by filtration, washed with diethyl ether, and dried under vacuum. ***Caution!**Perchlorate salts are potentially explosive and should be handled with care.* Electronic efficiency Ef = 1.0 mol F^–1^. Orange crystals suitable for X-ray diffraction studies of [Ag_2_(HL)_2_]·2CH_3_CN (3*) were obtained from the mother liquors of the synthesis.

The same procedure was followed for the synthesis of copper(I) mesocate **2** (5 mA and 9.5 V for 50 min; Ef = 1.1 mol F^–1^) and gold(I) mesocate **4** (5 mA and 9.5 V for 50 min; Ef = 0.9 mol F^–1^).

The proposed mechanism for their formation involves one electron for each metal atom as follows:
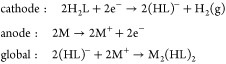


#### [Cu_2_(HL)_2_] (**2**)

Yield: 0.092 g (84%) of orange solid. The mp decomposes at 225 °C. Elem anal. Found: C, 65.2; H, 4.5; N, 8.0; S, 4.2. Calcd for C_78_H_62_N_8_P_4_S_2_Cu_2_: C, 65.8; H, 4.2; N, 7.9; S, 4.5. MALDI-TOF MS: *m*/*z* 713.1 ([Cu(HL) + H]^+^). ^1^H NMR (300 MHz, DMSO-*d*_6_): δ 11.78 (s, 1H, H_1_), 9.76 (s, 1H, H_1_), 8.56 (s, 2H, H_2_), 8.32–6.19 (m, H_Ar_). ^31^P NMR (300 MHz, DMSO-*d*_6_): δ 2.62, −1.71. IR (KBr, cm^–1^): ν(O–H + N–H) 3438 (f), ν(C–H) 3053 (d), ν(C=N + C–N) 1630 (d), 1500 (m), 1477 (f), 1435 (mf), ν(C=S) 1095 (m), 746 (m). Λ_M_ (μS cm^–1^): 4.0. UV–vis (λ_max_, nm): 358. Orange X-ray-quality crystals of [Cu_2_(HL)_2_]·3.5CH_3_CN (2*) were collected after filtration of the initial precipitate obtained during the synthesis, followed by slow evaporation of the mother liquors.

#### [Ag_2_(HL)_2_] (**3**)

Yield: 0.090 g (75%) of orange solid. The mp decomposes at 228 °C. Elem anal. Found: C, 61.5; H, 3.8; N, 7.3; S, 4.0. Calcd for C_78_H_62_N_8_P_4_S_2_Ag_2_: C, 61.9; H, 4.0; N, 7.4; S, 4.2. MALDI-TOF MS: *m*/*z* 757.0 ([Ag(HL) + H]^+^), 865.0 ([Ag_2_(HL)]^+^), 1515.0 ([Ag_2_(HL)_2_ + H]^+^). ^1^H NMR (300 MHz, DMSO-*d*_6_): δ 10.45 (s, 1H, H_1_), 9.04 (s, 1H, H_1_), 8.29 (s, 2H, H_2_), 7.99–6.58 (m, H_Ar_). ^31^P NMR (300 MHz, DMSO-*d*_6_): δ 6.19 (*J* = 364.1 Hz). IR (KBr, cm^–1^): ν(O–H + N–H) 3438 (mf), ν(C–H) 3053 (d), ν(C=N + C–N) 1631 (m), 1459 (d), 1435 (f), ν(C=S) 1095 (m), 746 (d). Λ_M_ (μS cm^–1^): 2.4. UV–vis (λ_max_, nm): 338. Yellow crystals suitable for X-ray diffraction studies of [Ag_2_(HL)_2_]·4CH_3_CN (3*) were obtained from the mother liquors of the synthesis.

#### [Au_2_(HL)_2_] (**4**)

Yield: 0.106 g (81%) of yellow solid. The mp decomposes at 228 °C. Elem anal. Found: C, 54.9; N, 6.7; H, 3.6; S, 3.6. Calcd for C_78_H_62_N_8_P_4_S_2_Au_2_: C, 55.4; N, 6.6; H, 3.6; S, 3.8. ESI^+^ MS: *m*/*z* 847.1 ([Au(HL) + H]^+^), 1693.3 ([Au_2_(HL)_2_]^+^). ^1^H NMR (300 MHz, DMSO-*d*_6_): δ 9.70 (s, 2H, H_1_), 9.02 (s, 2H, H_1_), 8.31 (s, 2H, H_2_), 7.89–6.52 (m, H_Ar_). ^31^P NMR (300 MHz, DMSO-*d*_6_): δ 34.57. IR (KBr, cm^–1^): ν(O–H + N–H) 3435 (mf), 3053 (d), ν(C–H) 2924 (d), ν(C=N + C–N) 1630 (m), 1510 (d), 1460 (f), 1435 (f), ν(C=S) 1097 (m), 748 (d). Λ_M_ (μS cm^–1^): 9.9. UV–vis (λ_max_, nm): 358, 408. Yellow crystals suitable for X-ray diffraction studies of [Au_2_(HL)_2_]·8CHCl_3_·C_6_H_14_ (4*) were obtained from recrystallization of the solid in a mixture of chloroform/hexane.

### Cationic Metal Complexes

Cationic metal complexes derived from copper(I), silver(I), and gold(I) salts were synthesized by the same procedure using acetonitrile as the solvent in PF_6_^–^ and BF_4_^–^ salts and methanol in the case of NO_3_^–^ and Cl^–^ salts. As an example, the synthetic procedure of complex [Cu_2_(H_2_L)_2_](PF_6_)_2_ (**5**) is summarized as follows: A total of 0.05 g of **1** (0.08 mmol) and 0.028 g of [Cu(CH_3_CN)_4_]PF_6_ (0.08 mmol) were mixed and dissolved in acetonitrile (50 mL). The resulting orange solution was refluxed for 3 h. Afterward, it was concentrated to a small volume (20 mL) and cooled overnight to 4 °C. The orange solid obtained was filtered off and washed with diethyl ether.

#### [Cu_2_(H_2_L)_2_](PF_6_)_2_ (**5**)

Yield: 0.106 g (78%) of orange solid. Mp: 265 °C. Elem anal. Found: C, 52.7; H, 3.9; N, 6.2; S, 3.6. Calcd for C_78_H_64_N_8_P_6_S_2_F_12_Cu_2_: C, 54.5; H, 3.8; N, 6.5; S, 3.7. ESI^+^ MS: *m*/*z*: 713.1 ([Cu(H_2_L)]^+^), 777.1 ([Cu_2_(H_2_L) – H]^+^), 1427.2 ([Cu_2_(H_2_L)_2_ – H]^+^). IR (KBr, cm^–1^): ν(NH) 3262, ν(C=N + C–N) 1585, 1545, 1479, ν(C=S) 1095, 748, ν(PF_6_) 845. Λ_M_ (μS cm^–1^): 138. UV–vis (λ_max_, nm): 346. Yellow X-ray-quality crystals of [Cu_2_(H_2_L)_2_](PF_6_)_2_·CH_3_CN·2H_2_O (5*) were collected after filtration of the initial precipitate obtained during the synthesis, followed by slow evaporation of the mother liquors.

#### [Cu_2_(H_2_L)_2_](BF_4_)_2_ (**6**)

Yield: 0.092 g (81%) of orange solid. Mp: 205 °C. Elem anal. Found: C, 57.3; H, 3.9; N, 6.7; S, 3.9. Calcd for C_78_H_64_N_8_P_4_S_2_B_2_F_8_Cu_2_: C, 58.5; H, 4.0; N, 7.0; S, 4.0. ESI^+^ MS: *m*/*z* 713.1 ([Cu(H_2_L)]^+^), 777.0 ([Cu_2_(H_2_L) – H]^+^), 1427.2 ([Cu_2_(H_2_L)_2_ – H]^+^). ^1^H NMR (300 MHz, CD_3_CN): δ 10.41 (s, 2H), 8.52 (s, 2H), 6.93 (s, 2H), 7.6–6.2 (H_Ar_). IR (KBr, cm^–1^): ν(NH) 3259, ν(C=N + C–N) 1631, 1541, 1435, ν(C=S) 1122, 750, ν(BF_4_) 1084. Λ_M_ (μS cm^–1^): 134. UV–vis (λ_max_, nm): 338. Orange X-ray-quality crystals of [Cu_2_(H_2_L)_2_](BF_4_)_2_·5CH_3_CN (6*) were grown by slow evaporation of the mother liquors from the synthesis.

#### [Ag_2_(H_2_L)_2_](PF_6_)_2_ (**7**)

Yield: 0.114 g (79%) of yellow solid. Mp: 225 °C. Elem anal. Found: C, 48.3; H, 3.1; N, 5.8; S, 3.2. Calcd for C_78_H_64_N_8_P_6_S_2_F_12_Ag_2_: C, 46.3; H, 3.2; N, 5.5; S, 3.2. ESI^+^ MS: *m*/*z*: 759.1 ([Ag(H_2_L)]^+^), 865.0 ([Ag_2_(H_2_L) – H]^+^), 1515.2 ([Ag_2_(H_2_L)_2_ – H]^+^), 1661.1 ({[Ag_2_(H_2_L)_2_]PF_6_}^+^). ^1^H NMR (300 MHz, CD_3_CN-*d*_3_): δ 9.79 (s, 2H), 8.66 (s, 2H), 7.68 (s, 2H), 6.94 (s, 2H), 7.8–6.4 (H_Ar_). IR (KBr, cm^–1^): ν(NH) 3258, ν(C=N + C–N) 1585, 1537, 1479, 1435, ν(C=S) 1095, 746, ν(PF_6_) 843. Λ_M_ (μS cm^–1^): 133. UV–vis (λ_max_, nm): 344. Yellow X-ray-quality crystals of [Ag_2_(H_2_L)_2_](PF_6_)_2_·6CH_3_CN (7*) were grown by slow evaporation of the mother liquors from the synthesis.

#### [Ag_4_(HL)_2_](NO_3_)_2_ (**8**)

Yield: 0.059 g (87%) of yellow solid. Mp: 205–210 °C. Elem anal. Found: C, 50.6; H, 3.7; N, 7.9; S, 3.4. Calcd for C_78_H_62_O_6_N_10_P_4_S_2_Ag_4_: C, 50.5; H, 3.4; N, 7.6; S, 3.5. ESI^+^ MS: *m*/*z* 865.0 ([Ag_2_(HL)]^+^), 1728.0 ([Ag_4_(HL)_2_ – H]^+^). ^1^H NMR (300 MHz, CD_3_CN): δ 10.02 (s, 1H), 8.84 (s, 1H), 8.25 (s, 2H), 8.15 (s, 2H), 7.8–6.3 (H_Ar_). IR (KBr, cm^–1^): ν(NH) 3262, ν(C=N + C–N) 1533, 1479, 1464, 1435, ν(N–O) 1385, ν(C=S) 1096, 748, ν(N–N) 1029. Λ_M_ (μS cm^–1^): 156. UV–vis (λ_max_, nm): 342. Colorless crystals suitable for X-ray diffraction studies of [Ag_4_(HL)_2_](NO_3_)_2_·4CH_3_OH (8*) were obtained from the mother liquors of the synthesis.

#### [Au_2_(H_2_L)_2_]Cl_2_ (**9**)

Yield: 0.081 g (78%) of yellow solid. Mp: 212 °C. Elem anal. Found: C, 53.0; H, 3.4; N, 6.2; S, 3.5. Calcd for Au_2_C_78_H_64_N_8_P_4_S_2_Cl_2_: C, 53.0; H, 3.8; N, 6.3; S, 3.6. ESI^+^ MS: *m*/*z* 847.2 ([Au(H_2_L)]^+^), 1043.1 ([Au_2_(H_2_L)]^+^), 1693.9 ([Au_2_(H_2_L)_2_ – H]^+^). IR (KBr, cm^–1^): ν(NH) 3251, ν(C=N + C–N) 1608, 1531, 1432, ν(C=S) 1117, 748. Λ_M_ (μS cm^–1^): 135. UV–vis (λ_max_, nm): 350. Yellow crystals suitable for X-ray diffraction studies of [Au_2_(H_2_L)_2_]Cl_2_·6.2CH_3_OH (9*) were obtained from the mother liquors of the synthesis.

## Supporting Information Available

Detailed experimental procedures, compound characterization, synthetic and crystallographic data, and Figures S1–S29 (PDF)

## References

[ref1] Song H.; Postings M.; Scott P.; Rogers N. J. Metallohelices Emulate the Properties o Short Cationic α-Helical Peptides. Chem. Sci. 2021, 12, 1620–1631. 10.1039/D0SC06412B. 34163922 PMC8179244

[ref2] Yang D.; Krbek L. K. S.; Yu L.; Ronson T. K.; Thoburn J. D.; Carpenter J. P.; Greenfield J. L.; Howe D. J.; Wu B.; Nitschke J. R. Glucose Binding Drives Reconfiguration of a Dynamic Library of Urea-Containing Metal-Organic Assemblies. Angew. Chem., Int. Ed. 2021, 60 (9), 4485–4490. 10.1002/anie.202014568. 33217126

[ref3] Barry D. E.; Kitchen J. A.; Pandurangan K.; Savyasachi A. J.; Peacock R. D.; Gunnlaugsson T. Formation of Enantiomerically Pure Luminescent Triple-Stranded Dimetallic Europium Helicates and Their Corresponding Hierarchical Self-Assembly Formation in Protic Polar Solutions. Inorg. Chem. 2020, 59 (5), 2646–2650. 10.1021/acs.inorgchem.0c00058. 32049514

[ref4] Kotova O.; Comby S.; Pandurangan K.; Stomeo F.; O'Brien J. E.; Feeney M.; Peacock R. D.; Mccoy C. P.; Gunnlaugsson T. The Effect of the Linker Size in C2-Symmetrical Chiral Ligands on the Self-Assembly Formation of Luminescent Triple-Stranded Di-Metallic Eu(III) Helicates in Solution. Dalton Trans. 2018, 47, 12308–12317. 10.1039/C8DT02753F. 30113616

[ref5] Casini A.; Woods B.; Wenzel M. The Promise of Self-Assembled 3D Supramolecular Coordination Complexes for Biomedical Applications. Inorg. Chem. 2017, 56, 14715–14729. 10.1021/acs.inorgchem.7b02599. 29172467

[ref6] Hannon M. J.; Childs L. J. Helices and Helicates: Beautiful Supramolecular Motifs with Emerging Applications. Supramol. Chem. 2004, 16 (1), 7–22. 10.1080/10610270310001632386.

[ref7] Albrecht M. Let’s Twist Again” - Double-Stranded, Triple-Stranded, and Circular Helicates. Chem. Rev. 2001, 101 (11), 3457–3497. 10.1021/cr0103672. 11840991

[ref8] Albrecht M.; Kotila S. Formation of a “Meso-Helicate” by Self-Assembly of Three Bis(Catecho1ate) Ligands and Two Titanium(1v) Ions. Angew. Chem., Int. Ed. Engl. 1995, 34 (19), 2134–2137. 10.1002/anie.199521341.

[ref9] Han G.; Zhou Y.; Yao Y.; Cheng Z.; Gao T.; Li H.; Yan P. Preorganized Helical Chirality Controlled Homochiral Self-Assembly and Circularly Polarized Luminescence of a Quadruple-Stranded Eu2 L 4 Helicate. Dalton Trans. 2020, 49 (10), 3312–3320. 10.1039/D0DT00062K. 32101214

[ref10] Hrabina O.; Malina J.; Kostrhunova H.; Novohradsky V.; Pracharova J.; Rogers N.; Simpson D. H.; Scott P.; Brabec V. Optically Pure Metallohelices That Accumulate in Cell Nuclei, Condense/Aggregate DNA, and Inhibit Activities of DNA Processing Enzymes. Inorg. Chem. 2020, 59 (5), 3304–3311. 10.1021/acs.inorgchem.0c00092. 32064865

[ref11] Xu Y. Y.; Sun O.; Qi Y.; Xie B. Y.; Gao T. Enhanced Luminescence for Detection of Small Molecules Based on Doped Lanthanide Compounds with a Dinuclear Double-Stranded Helicate Structure. New J. Chem. 2019, 43 (42), 16706–16713. 10.1039/C9NJ04104D.

[ref12] Vázquez M.; Taglietti A.; Gatteschi D.; Sorace L.; Sangregorio C.; González A. M.; Maneiro M.; Pedrido R. M.; Bermejo M. R. A 3D Network of Helicates Fully Assembled by π-Stacking Interactions. Chem. Commun. 2003, 3 (15), 1840–1841. 10.1039/B303549B. 12931993

[ref13] Mariano L. D. S.; Rosa I. M. L.; De Campos N. R.; Doriguetto A. C.; Dias D. F.; Do Pim W. D.; Valdo A. K. S. M.; Martins F. T.; Ribeiro M. A.; De Paula E. E. B.; Pedroso E. F.; Stumpf H. O.; Cano J.; Lloret F.; Julve M.; Marinho M. V. Polymorphic Derivatives of NiII and CoII Mesocates with 3D Networks and “Brick and Mortar” Structures: Preparation, Structural Characterization, and Cryomagnetic Investigation of New Single-Molecule Magnets. Cryst. Growth Des. 2020, 20 (4), 2462–2476. 10.1021/acs.cgd.9b01638.

[ref14] Palacios M. A.; Morlieras J.; Herrera J. M.; Mota A. J.; Brechin E. K.; Triki S.; Colacio E. Synthetic Ability of Dinuclear Mesocates Containing 1,3-Bis(Diazinecarboxamide)Benzene Bridging Ligands to Form Complexes of Increased Nuclearity. Crystal Structures, Magnetic Properties and Theoretical Studies. Dalton Trans. 2017, 46 (31), 10469–10483. 10.1039/C7DT02288C. 28752869

[ref15] Ferrando-Soria J.; Fabelo O.; Castellano M.; Cano J.; Fordham S.; Zhou H. C. Multielectron Oxidation in a Ferromagnetically Coupled Dinickel(II) Triple Mesocate. Chem. Commun. 2015, 51 (69), 13381–13384. 10.1039/C5CC03544A. 26207532

[ref16] Dul M. C.; Pardo E.; Lescouëzec R.; Chamoreau L. M.; Villain F.; Journaux Y.; Ruiz-García R.; Cano J.; Julve M.; Lloret F.; Pasán J.; Ruiz-Pérez C. Redox Switch-off of the Ferromagnetic Coupling in a Mixed-Spin Tricobalt(II) Triple Mesocate. J. Am. Chem. Soc. 2009, 131 (41), 14614–14615. 10.1021/ja9052202. 19778040

[ref17] Cangussu D.; Pardo E.; Dul M. C.; Lescouëzec R.; Herson P.; Journaux Y.; Pedroso E. F.; Pereira C. L. M.; Stumpf H. O.; Carmen Muñoz M.; Ruiz-García R.; Cano J.; Julve M.; Lloret F. Rational Design of a New Class of Heterobimetallic Molecule-Based Magnets: Synthesis, Crystal Structures, and Magnetic Properties of Oxamato-Bridged M3′ M2 (M′ = LiI and MnII; M = NiII and CoII) Open-Frameworks with a Three-Dimensional Honeycomb Architect. Inorg. Chim. Acta 2008, 361 (12–13), 3394–3402. 10.1016/j.ica.2008.02.042.

[ref18] Benito Q.; Balogh C. M.; El Moll H.; Gacoin T.; Cordier M.; Rakhmatullin A.; Latouche C.; Martineau-Corcos C.; Perruchas S. Luminescence Vapochromism of a Dynamic Copper Iodide Mesocate. Chem. Eur. J. 2018, 24 (71), 18868–18872. 10.1002/chem.201804377. 30259587

[ref19] Han Q.; Wang L.; Shi Z.; Xu C.; Dong Z.; Mou Z.; Liu W. Self-Assembly of Luminescent Lanthanide Mesocates as Efficient Catalysts for Transforming Carbon Dioxide into Cyclic Carbonates. Chem. Asian J. 2017, 12 (12), 1364–1373. 10.1002/asia.201700418. 28444696

[ref20] Custelcean R.; Bonnesen P. V.; Roach B. D.; Duncan N. C. Ion-Pair Triple Helicates and Mesocates Self-Assembled from Ditopic 2,2′-Bipyridine-Bis(Urea) Ligands and Ni(Ii) or Fe(Ii) Sulfate Salts. Chem. Commun. 2012, 48 (60), 7438–7440. 10.1039/c2cc33062h. 22659755

[ref21] Li X.; Wu J.; Wang L.; He C.; Chen L.; Jiao Y.; Duan C. Mitochondrial-DNA-Targeted IrIII -Containing Metallohelices with Tunable Photodynamic Therapy Efficacy in Cancer Cells. Angew. Chem., Int. Ed. Engl. 2020, 59 (16), 6420–6427. 10.1002/anie.201915281. 31970856

[ref22] González-Barcia L. M.; Fernández-Fariña S.; Rodríguez-Silva L.; Bermejo M. R.; González-Noya A. M.; Pedrido R. Comparative Study of the Antitumoral Activity of Phosphine-Thiosemicarbazone Gold(I) Complexes Obtained by Different Methodologies. J. Inorg. Biochem. 2020, 203, 110931 10.1016/j.jinorgbio.2019.110931. 31786438

[ref23] Allison S. J.; Cooke D.; Davidson F. S.; Elliott P. I. P.; Faulkner R. A.; Griffiths H. B. S.; Harper O. J.; Hussain O.; Owen-Lynch P. J.; Phillips R. M.; Rice C. R.; Shepherd S. L.; Wheelhouse R. T. Ruthenium-Containing Linear Helicates and Mesocates with Tuneable P53-Selective Cytotoxicity in Colorectal Cancer Cells. Angew. Chem. 2018, 130 (31), 9947–9952. 10.1002/ange.201805510. 29863754

[ref24] Romero M. J.; Martínez-Calvo M.; Maneiro M.; Zaragoza G.; Pedrido R.; González-Noya A. M. Selective Metal-Assisted Assembly of Mesocates or Helicates with Tristhiosemicarbazone Ligands. Inorg. Chem. 2019, 58 (1), 881–889. 10.1021/acs.inorgchem.8b02996. 30585726

[ref25] Niklas J. E.; Hiti E. A.; Wilkinson G. R.; Mayhugh J. T.; Gorden J. D.; Gorden A. E. V. Steric Control of Mesocate and Helicate Formation: Bulky Pyrrol-2-Yl Schiff Base Complexes of Zn2+. Inorg. Chim. Acta 2022, 529, 120653 10.1016/j.ica.2021.120653.

[ref26] Díaz D. E.; Llanos L.; Arce P.; Lorca R.; Guerrero J.; Costamagna J.; Aravena D.; Ferraudi G.; Oliver A.; Lappin A. G.; Lemus L. Steric and Electronic Factors Affecting the Conformation of Bimetallic CuI Complexes: Effect of the Aliphatic Spacer of Tetracoordinating Schiff-Base Ligands. Chem. Eur. J. 2018, 24 (52), 13839–13849. 10.1002/chem.201802290. 29935009

[ref27] Martínez-Calvo M.; Romero M. J.; Pedrido R.; González-Noya A. M.; Zaragoza G.; Bermejo M. R. Metal Self-Recognition: A Pathway to Control the Formation of Dihelicates and Mesocates. Dalton Trans. 2012, 41 (43), 13395–13404. 10.1039/c2dt31770b. 23007703

[ref28] Lim S. H.; Cohen S. M. Self-Assembled Supramolecular Clusters Based on Phosphines and Coinage Metals: Tetrahedra, Helicates, and Mesocates. Inorg. Chem. 2013, 52 (14), 7862–7872. 10.1021/ic302840x. 23799780

[ref29] Bullock S. J.; Davidson F. S.; Faulkner R. A.; Parkes G. M. B.; Rice C. R.; Towns-Andrews L. Control of Metallo-Supramolecular Assemblies via Steric, Hydrogen Bonding and Argentophilic Interactions; Formation of a 3-Dimensional Polymer of Circular Helicates. CrystEngComm 2017, 19 (9), 1273–1278. 10.1039/C6CE02589G.

[ref30] Argent S. P.; Adams H.; Riis-Johannessen T.; Jeffery J. C.; Harding L. P.; Clegg W.; Harrington R. W.; Ward M. D. Complexes of Ag(i), Hg(i) and Hg(Ii) with Multidentate Pyrazolyl-Pyridine Ligands: From Mononuclear Complexes to Coordination Polymers via Helicates, a Mesocate, a Cage and a Catenate. J. Chem. Soc., Dalton Trans. 2006, 42, 4996–5013. 10.1039/b607541j. 17060986

[ref31] Fielden J.; Long D.; Evans C.; Cronin L. Metal-Dependent Formation of Mononuclear Complexes and M 2 L 2 Mesocates with Schiff-Base Ligands. Eur. J. Inorg. Chem. 2006, 2006, 3930–3935. 10.1002/ejic.200600367.

[ref32] Casas J. S.; García-Tasende M. S.; Sordo J. Main Group Metal Complexes of Semicarbazones and Thiosemicarbazones. A Structural Review. Coord. Chem. Rev. 2000, 209, 197–261. 10.1016/S0010-8545(00)00363-5.

[ref33] Siddiqui E. J.; Azad I.; Khan A. R.; Khan T. Thiosemicarbazone Complexes as Versatile Medicinal Chemistry Agents: A Review. J. Drug Delivery Ther. 2019, 9 (3), 689–703. 10.22270/jddt.v9i3.2888.

[ref34] Bonaccorso C.; Marzo T.; La Mendola D. Biological Applications of Thiocarbohydrazones and Their Metal Complexes: A Perspective Review. Pharmaceuticals 2020, 13 ( (1), ). 10.3390/ph13010004 PMC7169414 31881715

[ref35] Dragancea D.; Arion V. B.; Shova S.; Rentschler E.; Gerbeleu N. V. Azine-Bridged Octanuclear Copper(II) Complexes Assembled with a One-Stranded Ditopic Thiocarbohydrazone Ligand. Angew. Chem. 2005, 117 (48), 8152–8156. 10.1002/ange.200501807. 16287190

[ref36] Tandon S. S.; Dul M. C.; Lee J. L.; Dawe L. N.; Anwar M. U.; Thompson L. K. Complexes of Ditopic Carbo- and Thio-Carbohydrazone Ligands - Mononuclear, 1D Chain, Dinuclear and Tetranuclear Examples. Dalton Trans. 2011, 40 (14), 3466–3475. 10.1039/c0dt01487g. 21347464

[ref37] Kaya Y.; Erçaǧ A.; Koca A. New Square-Planar Nickel(II)-Triphenylphosphine Complexes Containing ONS Donor Ligands: Synthesis, Characterization, Electrochemical and Antioxidant Properties. J. Mol. Struct. 2020, 1206, 127653 10.1016/j.molstruc.2019.127653.

[ref38] Zafarian H.; Sedaghat T.; Motamedi H.; Amiri Rudbari H. A Multiprotic Ditopic Thiocarbohydrazone Ligand in the Formation of Mono- and Di-Nuclear Organotin(IV) Complexes: Crystal Structure, Antibacterial Activity and DNA Cleavage. J. Organomet. Chem. 2016, 825–826, 25–32. 10.1016/j.jorganchem.2016.10.023.

[ref39] Qiao C. J.; Ding D.; Li J.; Gu L.; Xu Y.; Fan Y. T. Synthesis, Crystal Structure and Electrochemical Properties of a Novel Tetranuclear Silver(I) Cluster Based on 1,1′-Bis[(2-Hydroxybenzylidene)Thiocarbonohydrazono-1-Ethyl] Ferrocene (FcL). Inorg. Chem. Commun. 2009, 12 (10), 1057–1060. 10.1016/j.inoche.2009.08.020.

[ref40] Ibrahim A. A.; Khaledi H.; Ali H. M. A Multiprotic Indole-Based Thiocarbohydrazone in the Formation of Mono-, Di- and Hexa-Nuclear Metal Complexes. Polyhedron 2014, 81, 457–464. 10.1016/j.poly.2014.06.057.

[ref41] Martínez-Calvo M.; Pedrido R.; González-Noya A. M.; Romero M. J.; Cwiklinska M.; Zaragoza G.; Bermejo M. R. A Sequentially Assembled Grid Composed of Supramolecular Meso-Helical Nodes. Chem. Commun. 2011, 47 (34), 9633–9635. 10.1039/c1cc13582a. 21808784

[ref42] Fernández-Fariña S.; González-Barcia L. M.; Romero M. J.; García-Tojal J.; Maneiro M.; Seco J. M.; Zaragoza G.; Martínez-Calvo M.; González-Noya A. M.; Pedrido R. Conversion of a Double-Tetranuclear Cluster Silver Helicate into a Dihelicate via a Rare Desulfurization Process. Inorg. Chem. Front. 2022, 9, 531 10.1039/D1QI01308D.

[ref43] Bermejo M. R.; González-Noya A. M.; Martínez-Calvo M.; Pedrido R.; Romero M. J.; Vázquez López M. Checking the Route to Cluster Helicates. Eur. J. Inorg. Chem. 2008, 2008, 3852–3863. 10.1002/ejic.200800477.

[ref44] Bermejo M. R.; González-Noya A. M.; Pedrido R. M.; Romero M. J.; Vázquez M. Route to Cluster Helicates. Angew. Chem., Int. Ed. 2005, 44 (27), 4182–4187. 10.1002/anie.200500840. 15929158

[ref45] Rodríguez A.; García-vázquez J. A. The Use of Sacrificial Anodes for the Electrochemical Synthesis of Metallic Complexes. Coord. Chem. Rev. 2015, 303, 42–85. 10.1016/j.ccr.2015.05.006.

[ref46] González-Barcia L. M.; Romero M. J.; González Noya A. M.; Bermejo M. R.; Maneiro M.; Zaragoza G.; Pedrido R. the Golden Method”: Electrochemical Synthesis Is an Efficient Route to Gold Complexes. Inorg. Chem. 2016, 55 (16), 7823–7825. 10.1021/acs.inorgchem.6b01362. 27483164

[ref47] Geary W. J. The Use of Conductivity Measurements in Organic Solvents for the Characterisation of Coordination Compounds. Coord. Chem. Rev. 1971, 7 (1), 81–122. 10.1016/S0010-8545(00)80009-0.

[ref48] Ohkouchi M.; Masui D.; Yamaguchi M.; Yamagishi T. Mechanism of Silver(I)-Catalyzed Mukaiyama Aldol Reaction: Active Species in Solution in AgPF6-(S)-BINAP versus AgOAc-(S)-BINAP Systems. J. Mol. Catal. Chem. 2001, 170 (1–2), 1–15. 10.1016/S1381-1169(01)00049-8.

[ref49] Castiñeiras A.; Pedrido R. Aurophilicity in Gold(I) Thiosemicarbazone Clusters. Dalton Trans. 2012, 41 (4), 1363–1372. 10.1039/C1DT11680K. 22113610

[ref50] Ilie A.; Raţ C. I.; Scheutzow S.; Kiske C.; Lux K.; Klapötke T. M.; Silvestru C.; Karaghiosoff K. Metallophilic Bonding and Agostic Interactions in Gold(I) and Silver(I) Complexes Bearing a Thiotetrazole Unit. Inorg. Chem. 2011, 50 (6), 2675–2684. 10.1021/ic102595d. 21309537

[ref51] Ali Kamyabi M.; Alirezaei F.; Soleymani-Bonoti F.; Bikas R.; Siczek M.; Lis T. Efficient Reduction of Dioxygen with Ferrocene Catalyzed by Thiocarbohydrazone Tetranuclear Cobalt(III) Coordination Compound. Appl. Organomet. Chem. 2020, e5833 10.1002/aoc.5833.

[ref52] Dragancea D.; Addison A. W.; Zeller M.; Thompson L. K.; Hoole D.; Revenco M. D.; Hunter A. D. Dinuclear Copper(II) Complexes with Bis-Thiocarbohydrazone Ligands. Eur. J. Inorg. Chem. 2008, 2008, 2530–2536. 10.1002/ejic.200701187.

[ref53] Wells A. F. Structural Inorganic Chemistry, 5rd ed.; Clarendon Press: London, 1990.

[ref54] Bondi A. Van Der Waals Volumes and Radii. J. Phys. Chem. 1964, 68 (3), 441–451. 10.1021/j100785a001.

[ref55] Vázquez López M.; Zaragoza G.; Pedrido R.; Rama G.; Bermejo M. R. Delving into the Second Supramolecular Event Approach: Aggregation of Small Metallo-Supramolecular Units Supported by One or Two Types of Non-Covalent Forces. Inorg. Chem. Commun. 2008, 11 (9), 995–998. 10.1016/j.inoche.2008.04.039.

[ref56] Castiñeiras A.; Pedrido R. Factors Involved in the Nuclearity of Silver Thiosemicarbazone Clusters: Cocrystallization of Two Different Sized Tetranuclear Silver(I) Clusters Derived from a Phosphinothiosemicarbazone Ligand. Inorg. Chem. 2008, 47 (13), 5534–5536. 10.1021/ic800178v. 18533649

