# Supplementary material for: Two Synthetic Approaches to Coinage Metal(I) Mesocates: Electrochemical versus Chemical Synthesis
Source: Inorg Chem. 2022 Aug 19;61(35):14121–30. doi: 10.1021/acs.inorgchem.2c02243 (PMC9455603; doi:10.1021/acs.inorgchem.2c02243)
Supplement: Supplementary file 1 — ic2c02243_si_001.pdf [file ic2c02243_si_001.pdf]

Supporting Information

Two synthetic approaches to coinage M(I) mesocates: electrochemical *versus* chemical synthesis

Sandra Fernández-Fariña,[a] Miguel Martínez-Calvo,[a] Marcelino Maneiro,[b] José M. Seco,[c] Guillermo Zaragoza,[d] Ana M. González-Noya[a,*] and Rosa Pedrido[a,*]

[a] *Departamento de Química Inorgánica, Facultade de Química, Campus Vida, Universidade de Santiago de Compostela, 15782 Santiago de Compostela, Spain.*

[b] *Departamento de Química Inorgánica, Facultade de Ciencias, Campus Terra, Universidade de Santiago de Compostela, 27002 Lugo, Spain.*

[c] *Departamento de Química Orgánica, Facultade de Química, Campus Vida, Universidade de Santiago de Compostela, 15782 Santiago de Compostela, Spain.*

[d] *Unidade de Difracción de Raios X, Edificio Cactus, Campus Vida, Universidade de Santiago de Compostela, E-15782, Santiago de Compostela, Spain.*

Table of contents

1. Electrochemical methodology 4

2. Thiocarbohydrazone ligand H_2L 1 5

Figure S1. Infrared spectrum of the ligand H_2L **1**. 5

Figure S2. ESI+ spectrum of the ligand H_2L 5

Figure S3. ^{1}H NMR spectrum of the ligand H_2L **1** (acetone-d_6, 278 K). 6

Figure S4. ^{1}H NMR spectrum of the ligand H_2L **1** (DMSO-d_6, 298 K)....................6

Figure S5. ^{13}C NMR spectrum of the ligand H_2L **1** (DMSO-d_6, 298 K). 7

Figure S6. ^{31}P NMR (DMSO-d_6) spectrum of the ligand H_2L **1** (DMSO-d_6, 298 K). 7

3. Neutral mesocates $[Cu_2(HL)_2]$ 2, $[Ag_2(HL)_2]$ 3 and $[Au_2(HL)_2]$ 4 8

Figure S7. Mass spectrum of the mesocate $[Ag_2(HL)_2]$ **3**, as an example of neutral complexes (**2**-**4**). 8

Figure S8. Superposition of the infrared spectra of the H_2L ligand **1** (dashed line, green) and the $[Cu_2(HL)_2]$ **2** mesocate (solid line, blue), as an example of neutral complexes (**2**-**4**). 8

Figure S9. ^{1}H NMR spectra of the complexes **2-4** (DMSO-d_6, 298 K). 9

Figure S10. ^{31}P NMR spectra of the complexes **2**-**4** (DMSO-d_6, 298 K). 10

Table S1. Main UV-vis absorption bands of the H_2L ligand and neutral mesocates (**2**-**4**) in acetonitrile. 10

Figure S11. Metallomacrocycle ring present in $[Cu_2(HL)_2]\cdot 3.5CH_3CN$ **2***. 11

Figure S12. Metallomacrocycle ring present in $[Ag_2(HL)_2]\cdot 4CH_3CN$ **3***. 11

Figure S13. Metallomacrocycle ring present in $[Au_2(HL)_2]\cdot 8CHCl\cdot C_6H_{14}$ **4***.......11

Figure S14. Intramolecular pseudo-agostic interactions [CH(29)···Ag1 2.95 Å, NH(2)···Ag1 3.01 Å] in $[Ag_2(HL)_2]\cdot 4CH_3CN$ **3***. 12

Figure S15. Intramolecular pseudo-agostic interactions [CH(33)···Au1 3.013 Å, CH(21)···Au1 2.97 Å] in $[Au_2(HL)_2]\cdot 8CHCl\cdot C_6H_{14}$ **4***. 12

Figure S16. Intramolecular CH-π interactions for $[Cu_2(HL)_2]\cdot 3.5CH_3CN$ **2***........13

4. Bideprotonated copper complex $[Cu_4L_2]$ 13

Figure S17. Superposition of the infrared spectra of $[Cu_4L_2]$ complex obtained in bideprotonation conditions (dashed line, green) and $[Cu_2(HL)_2]$ **2** complex (solid line, violet)..13

Figure S18. Mass spectrum of the tetranuclear copper complex, $[Cu_4(L)_2]$. 14

5. Cationic mesocates $[Cu_2(H_2L)_2](PF_6)_2$ 5, $[Cu_2(H_2L)_2](BF_4)_2$ 6, $[Ag_2(H_2L)_2](PF_6)_2$ 7, $[Ag_4(HL)_2](NO_3)_2$ 8, and $[Au_2(H_2L)_2]Cl_2$ 9 14

Figure S19. Superposition of the infrared spectra of the H_2L ligand **1** (dashed line, blue) and the $[Cu_2(H_2L)_2](PF_6)_2$ **5** mesocate (solid line, orange). 14

Figure S20. Superposition of the infrared spectra of the H_2L ligand **1** (dashed line, blue) and the $[Cu_2(H_2L)_2](BF_4)_2$ **6** mesocate (solid line, green). 15

Figure S21. Superposition of the infrared spectra of the H_2L ligand **1** (dashed line, blue) and the $[Ag_2(H_2L)_2](PF_6)_2$ **7** mesocate (solid line, violet). 15

Figure S22. Superposition of the infrared spectra of the H_2L ligand **1** (dashed line, blue) and the $[Ag_4(HL)_2](NO_3)_2$ **8** mesocate (solid line, pink). 16

Figure S23. Mass spectrum of the $[Ag_2(H_2L)_2](PF_6)_2$ **7** mesocate.........................16
Figure S24. Mass spectrum of the $[Ag_4(HL)_2](NO_3)_2$ **8** mesocate..........................17
Figure S25. ^{1}H NMR spectrums of the copper(I) cationic mesocates **5** and **6** (CD_3CN-d_3, 298 K). 17
Figure S26. ^{1}H NMR spectrums of the silver(I) cationic mesocates **7** and **8** (CD_3CN-d_3, 298 K). 18
Figure S27. Superposition of the UV-Vis absorption spectra of the ligand and the ionic complexes **5-9** (CD_3CN-d_3, 298 K). 18
Figure S28. Sticks diagram of the dicationic copper(I) mesocate $[Cu_2(H_2L)_2](PF_6)_2·7CH_3OH$ **5***. 19
Figure S29. Sticks diagram of the dicationic copper(I) mesocate $[Cu_2(H_2L)_2](BF_4)_2·5CH_3CN·$**6***. 19

6. **Crystallographic data of mesocates 2*–9* 20**
Table S2. Main bond distances (Å) and angles (°) in $[Cu_2(HL)_2]·3.5CH_3CN$ **2***...23
Table S3. Main bond distances (Å) and angles (°) in $[Ag_2(HL)_2]·4CH_3CN$ **3***......23
Table S4. Main bond distances (Å) and angles (°) in $[Au_2(HL)_2]·8CHCl_3·C_6H_{14}$ **4***. 23
Table S5. Main bond distances (Å) and angles (°) in $[Cu_2(H_2L)_2](BF_4)_2·CH_3CN·2H_2O$ **5***..........24
Table S6. Main bond distances (Å) and angles (°) in $[Cu_2(H_2L)_2](BF_4)_2·5CH_3CN·$**6***. 24
Table S7. Main bond distances (Å) and angles (°) in $[Ag_2(H_2L)_2](PF_6)_2·6CH_3CN·$**7***. 24
Table S8. Main bond distances (Å) and angles (°) in $[Ag_4(HL)_2](NO_3)_2·4CH_3OH·$**8***. 25
Table S9. Main bond distances (Å) and angles (°) in $[Au_2(H_2L)_2]Cl_2·7CH_3OH$ **9***. 25

7. **References 26**

1. Electrochemical methodology

In order to carry out an electrochemical synthesis a power supply to control the intensity and potential, at which the reaction takes place, and an electrochemical cell are required.

The electrochemical cell contains a solution of the corresponding ligand in the solvent used and a small amount of tetraethylammonium perchlorate which acts as conductive electrolyte. During the course of the electrochemical reaction, reduction of the ligand takes place at the platinum cathode, while oxidation will take place at the metal anode, which will supply the metal ions necessary to form the metallo-supramolecular complex.

The electrochemical cell can be represented as:

$$Pt(-) \mid \text{ligand + solvent} \mid M(+)$$

The proposed mechanism for the formation of the neutral mesocates **2-4** $[M_2(HL)_2]$ involves one electron per ligand, as shown below:

$$\text{Cathode: } 2\ H_2L + 2\ e^- \rightarrow 2\ (HL)^- + H_2(g)$$

$$\text{Anode: } 2\ M \rightarrow 2\ M^+ + 2\ e^-$$

$$\text{Global: } 2\ (HL)^- + 2\ M^+ \rightarrow M_2(HL)_2$$

The proposed mechanism for the formation of the bideprotonated compound $[Cu_4L_2]$ involves two electrons for ligand, as shown below:

$$\text{Cathode: } 2\ H_2L + 4\ e^- \rightarrow 2\ (L)^- + H_2(g)$$

$$\text{Anode: } 4\ M \rightarrow 4\ M^+ + 4\ e^-$$

$$\text{Global: } 2\ (L)^- + 4\ M^+ \rightarrow M_4(L)_2$$

2. Thiocarbohydrazone ligand H_2L 1

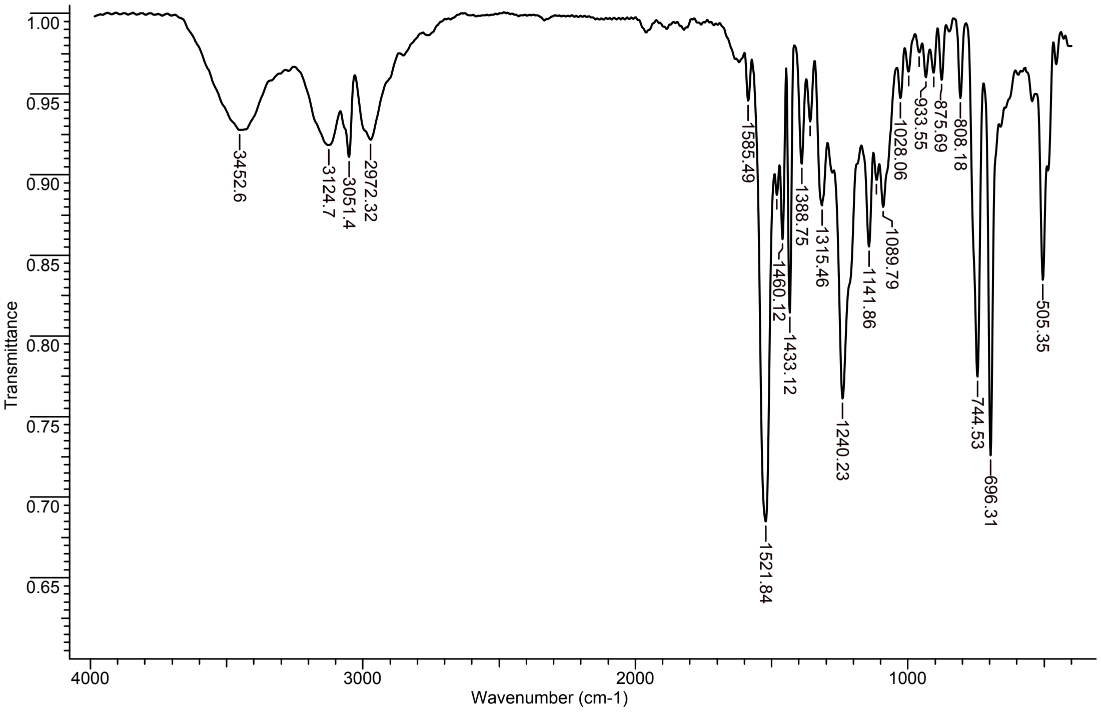

Figure S1. Infrared spectrum of the ligand H_2L **1**.

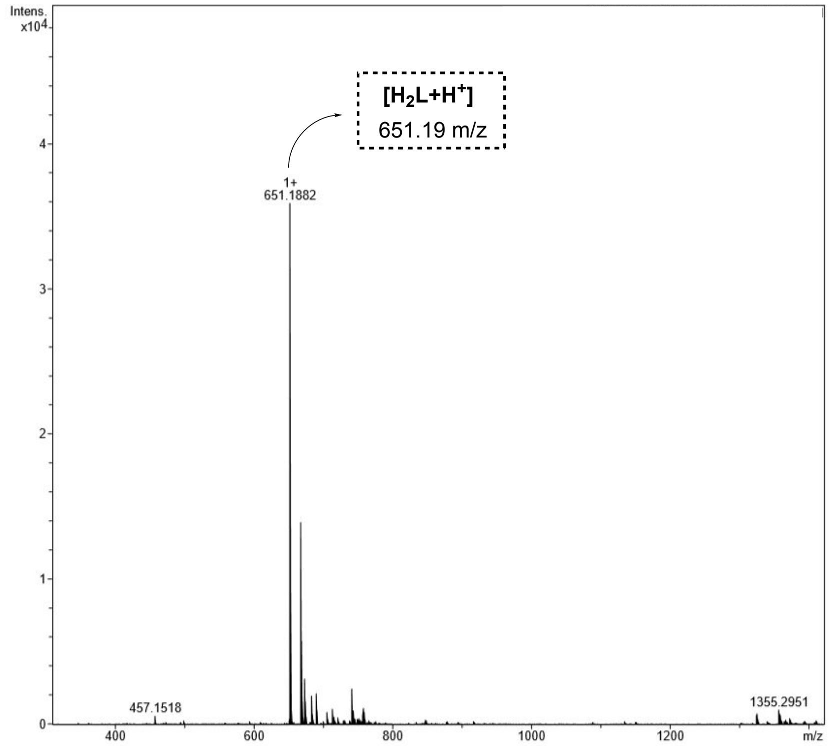

Figure S2. ESI+ spectrum of the ligand H_2L **1**.

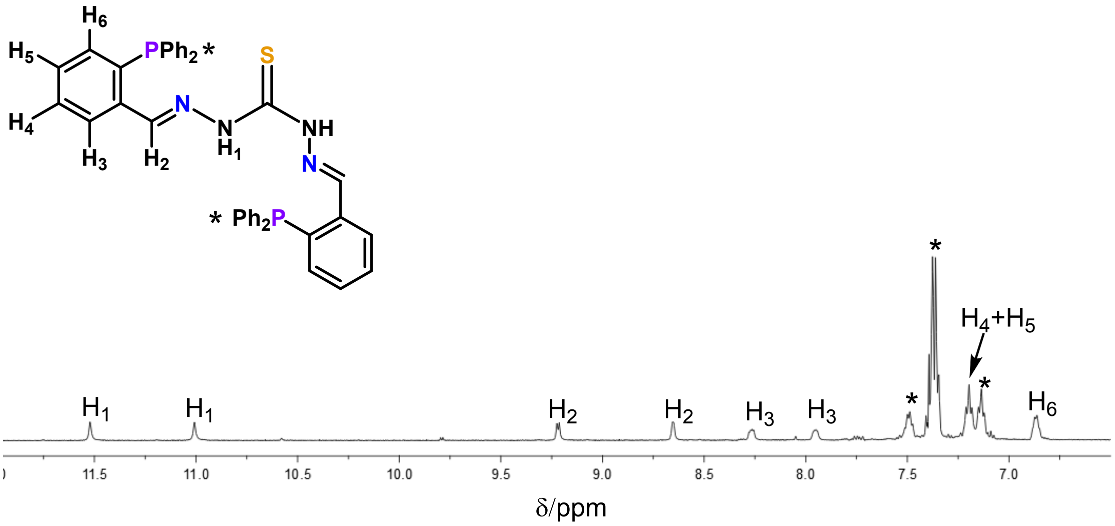

Figure S3. ^{1}H NMR spectrum of the ligand H_2L **1** (acetone-d_6, 278 K).

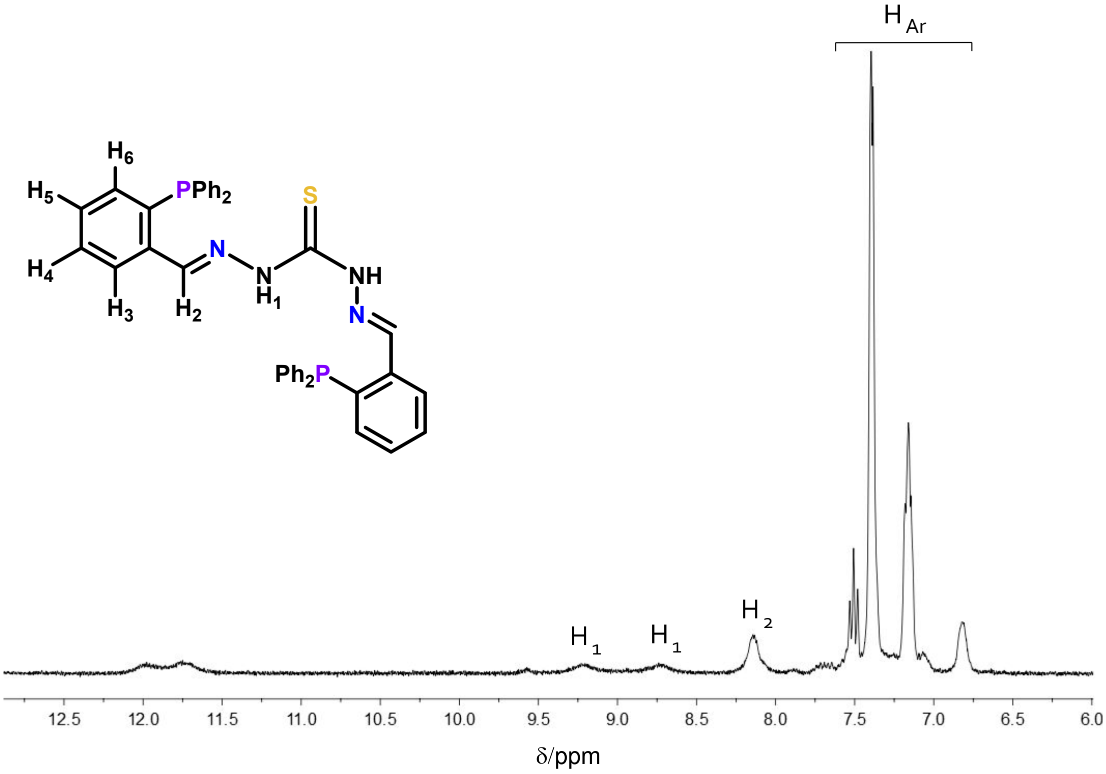

Figure S4. ^{1}H NMR spectrum of the ligand H_2L **1** (DMSO-d_6, 298 K).

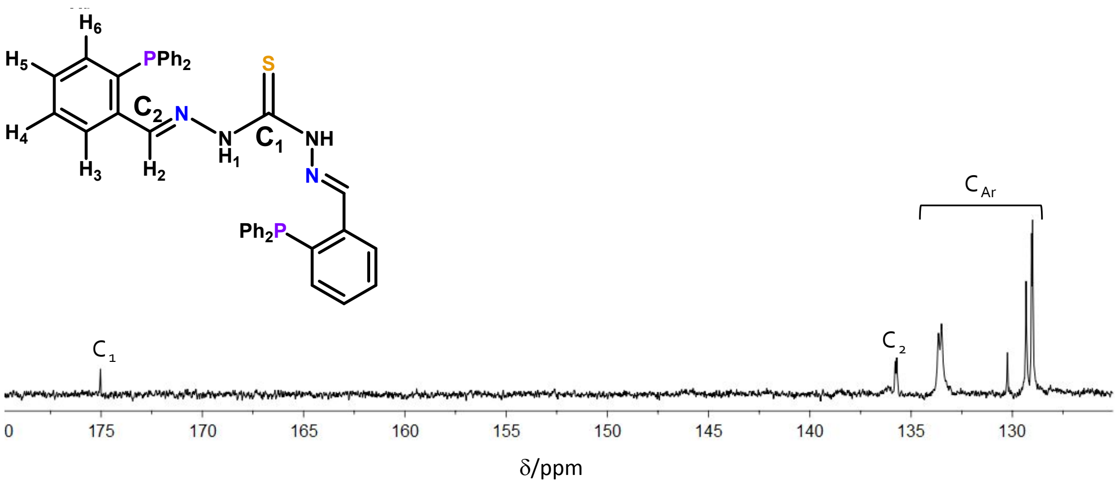

Figure S5. ^{13}C NMR spectrum of the ligand H_2L **1** (DMSO-d_6, 298 K).

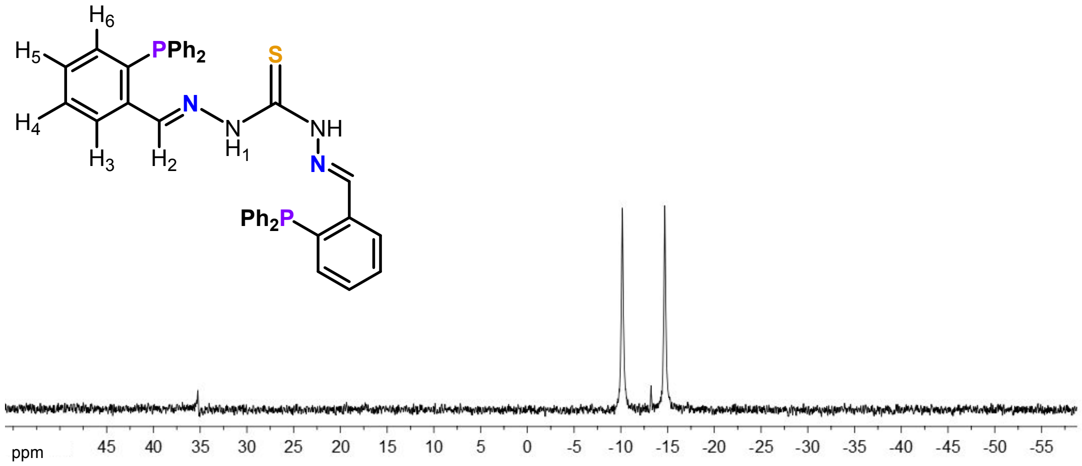

Figure S6. ^{31}P NMR (DMSO-d_6) spectrum of the ligand H_2L **1** (DMSO-d_6, 298 K).

3. Neutral mesocates [$Cu_2(HL)_2$] 2, [$Ag_2(HL)_2$] 3 and [$Au_2(HL)_2$] 4

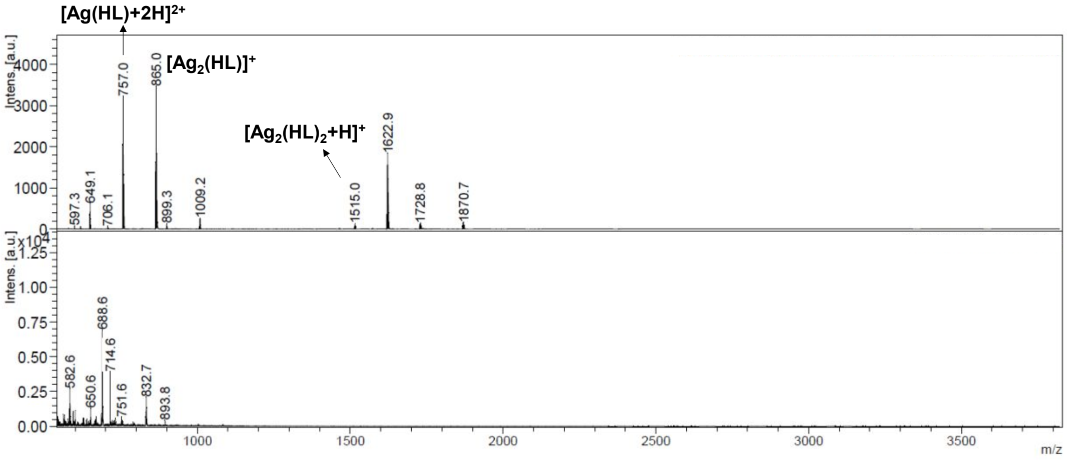

Figure S7. Mass spectrum of the mesocate [$Ag_2(HL)_2$] **3**, as an example of neutral complexes (**2-4**).

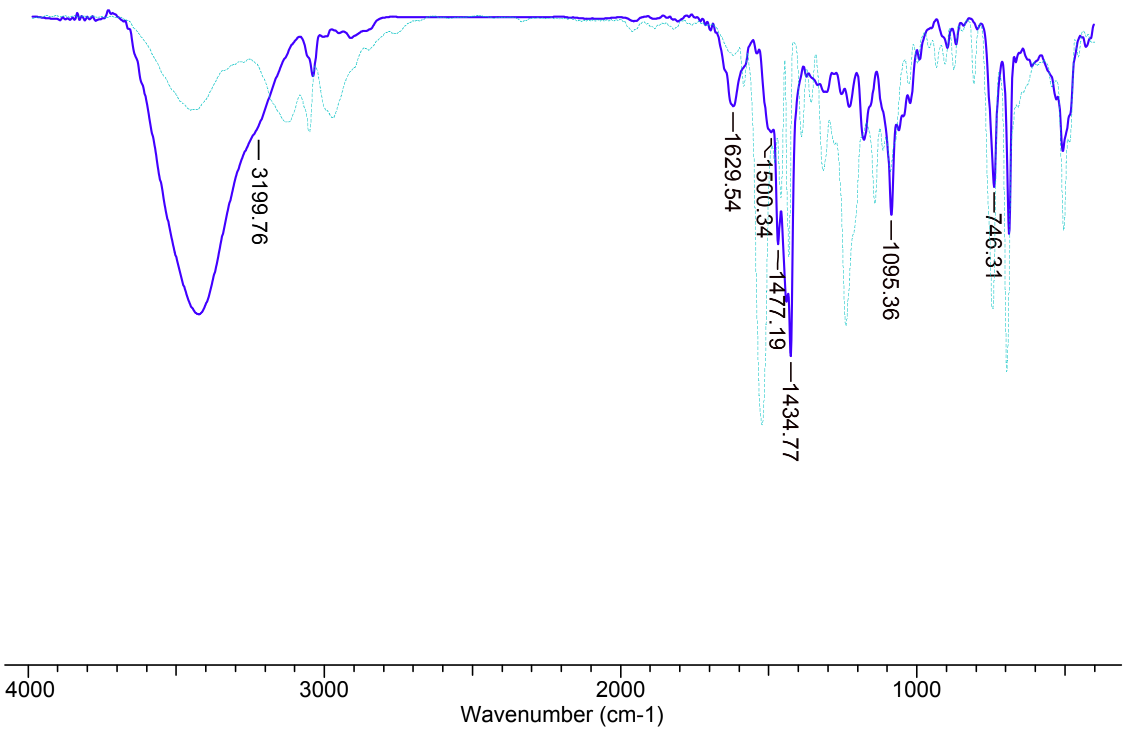

Figure S8. Superposition of the infrared spectra of the H_2L ligand **1** (dashed line, green) and the $[Cu_2(HL)_2]$ **2** mesocate (solid line, blue), as an example of neutral complexes (**2-4)**.

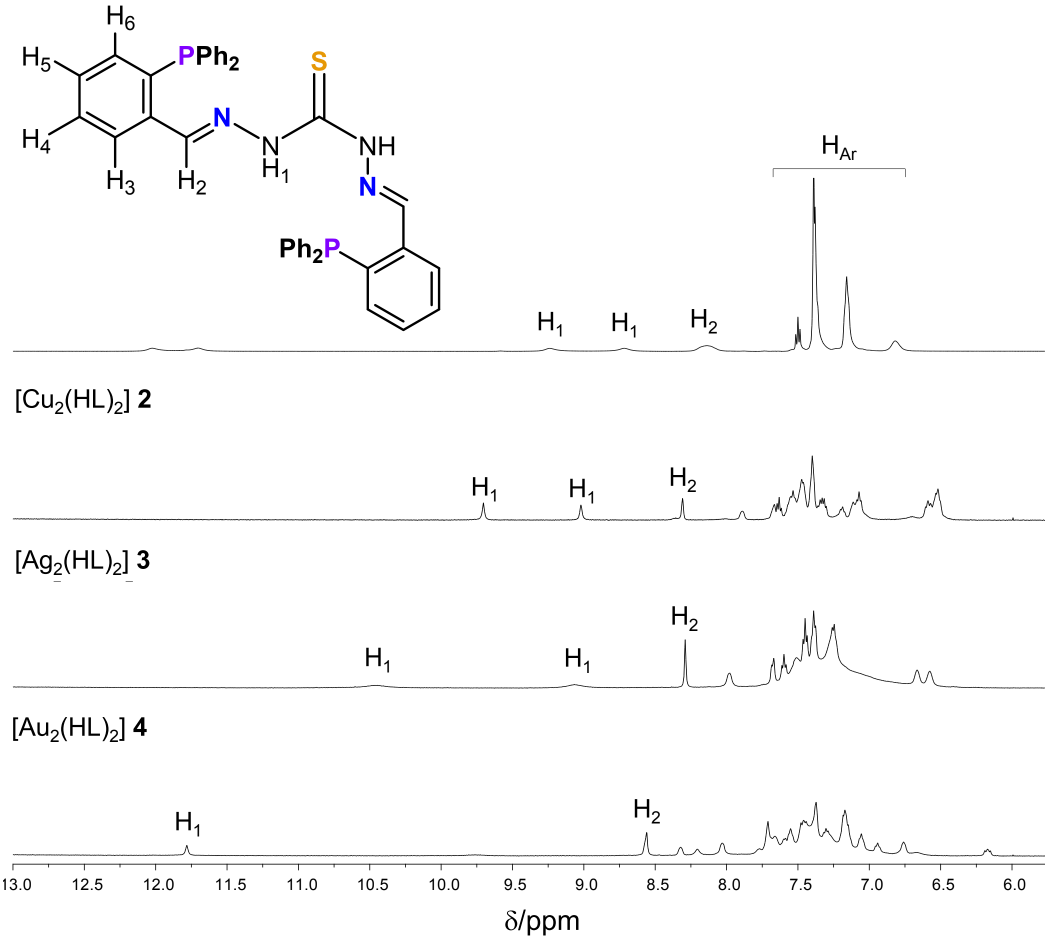

Figure S9. ^{1}H NMR spectra of the complexes **2-4** (DMSO-d_6, 298 K).

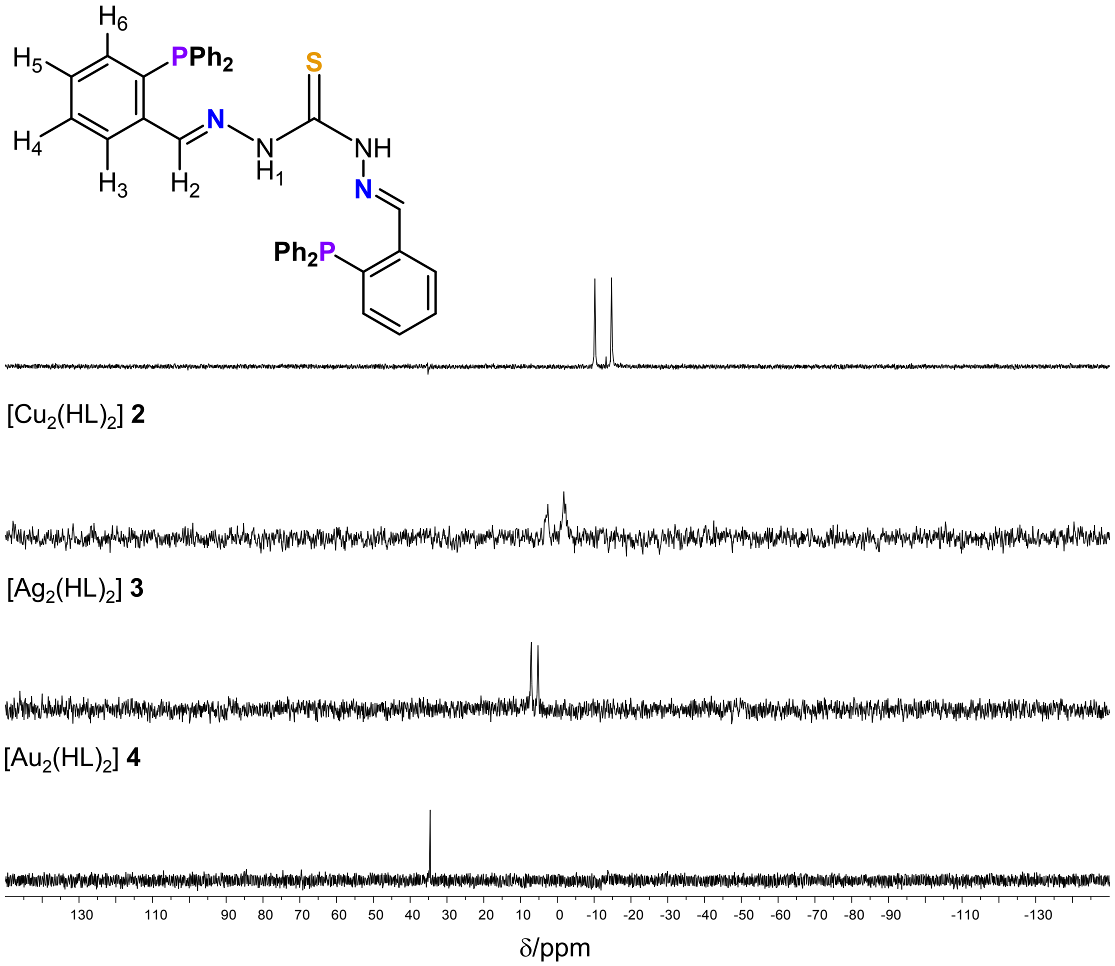

Figure S10. ^{31}P NMR spectra of the complexes **2-4** (DMSO-d_6, 298 K).

**Compound**	**λ_{max}/nm**
**H_2L**	276, 332
**$[Cu_2(HL)_2]$ 2**	358
**$[Ag_2(HL)_2]$ 3**	338
**$[Au_2(HL)_2]$ 4**	338, 400 (sh)

Table S1. Main UV-vis absorption bands of the H_2L ligand and neutral mesocates (**2-4**) in acetonitrile.

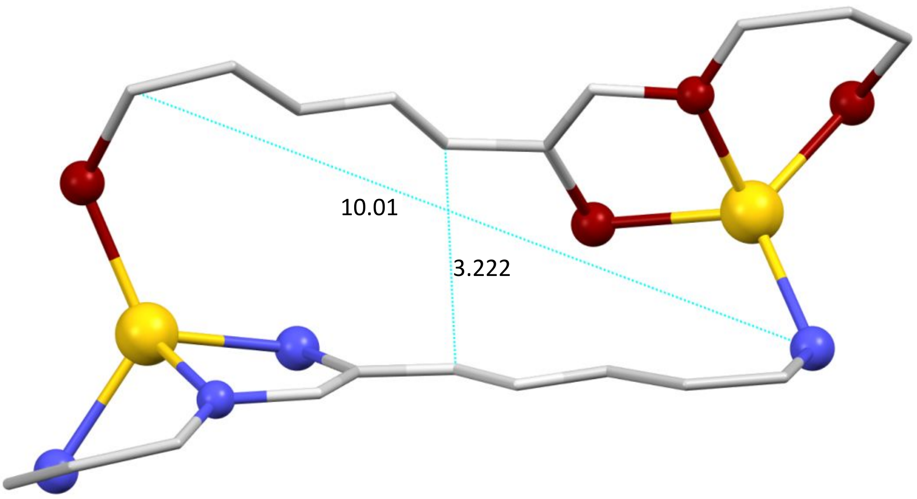

Figure S11. Metallomacrocycle ring present in $[Cu_2(HL)_2]\cdot 3.5CH_3CN$ **2***.

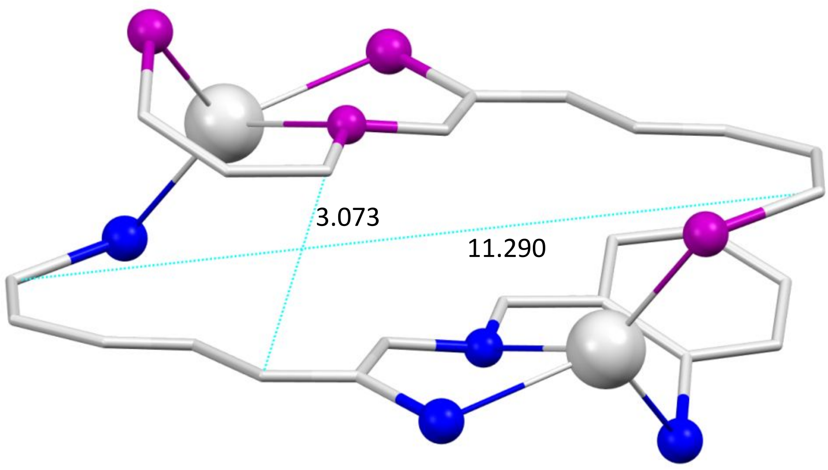

Figure S12. Metallomacrocycle ring present in $[Ag_2(HL)_2]\cdot 4CH_3CN$ **3***.

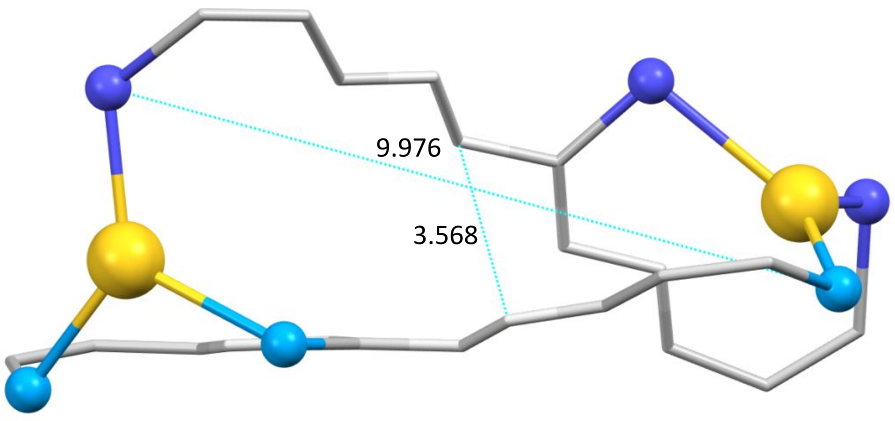

Figure S13. Metallomacrocycle ring present in $[Au_2(HL)_2]\cdot 8CHCl\cdot C_6H_{14}$ **4***.

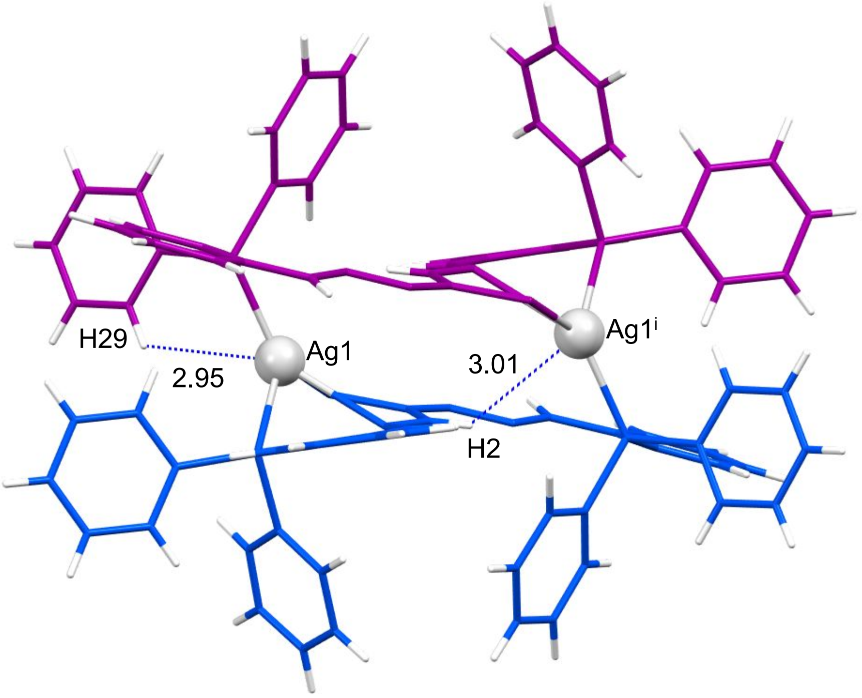

Figure S14. Intramolecular pseudo-agostic interactions [CH(29)···Ag1 2.95 Å, NH(2)···Ag1 3.01 Å] in $[Ag_2(HL)_2]\cdot 4CH_3CN$ **3***.

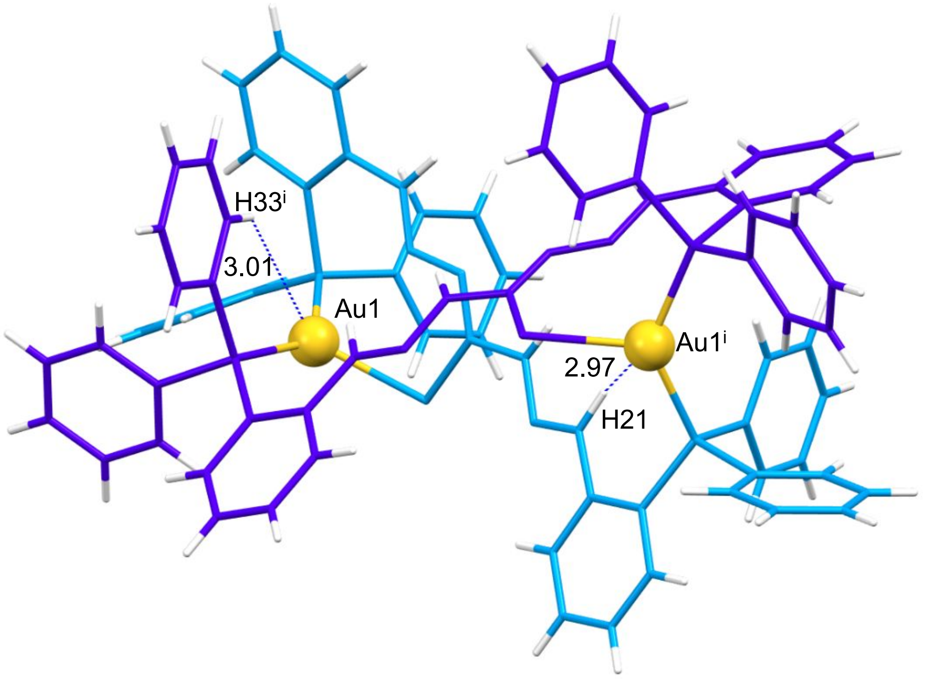

Figure S15. Intramolecular pseudo-agostic interactions [CH(33)···Au1 3.013 Å, CH(21)···Au1 2.97 Å] in $[Au_2(HL)_2]\cdot 8CHCl\cdot C_6H_{14}$ **4***.

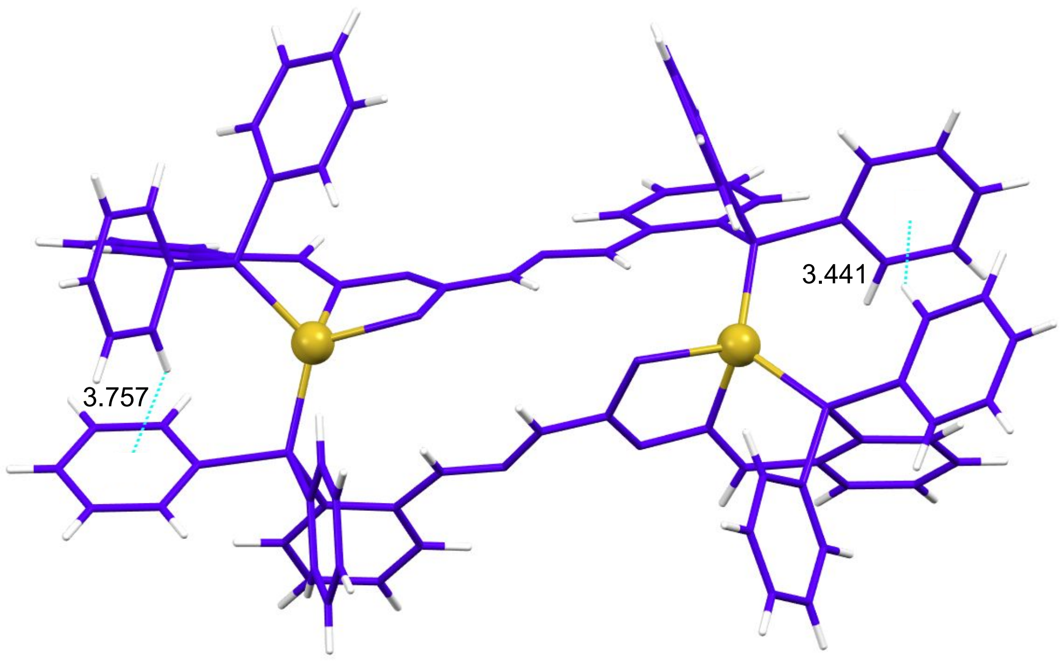

Figure S16. Intramolecular CH-π interactions for $[Cu_2(HL)_2]\cdot3.5CH_3CN$ **2***.

4. Bideprotonated copper complex $[Cu_4L_2]$

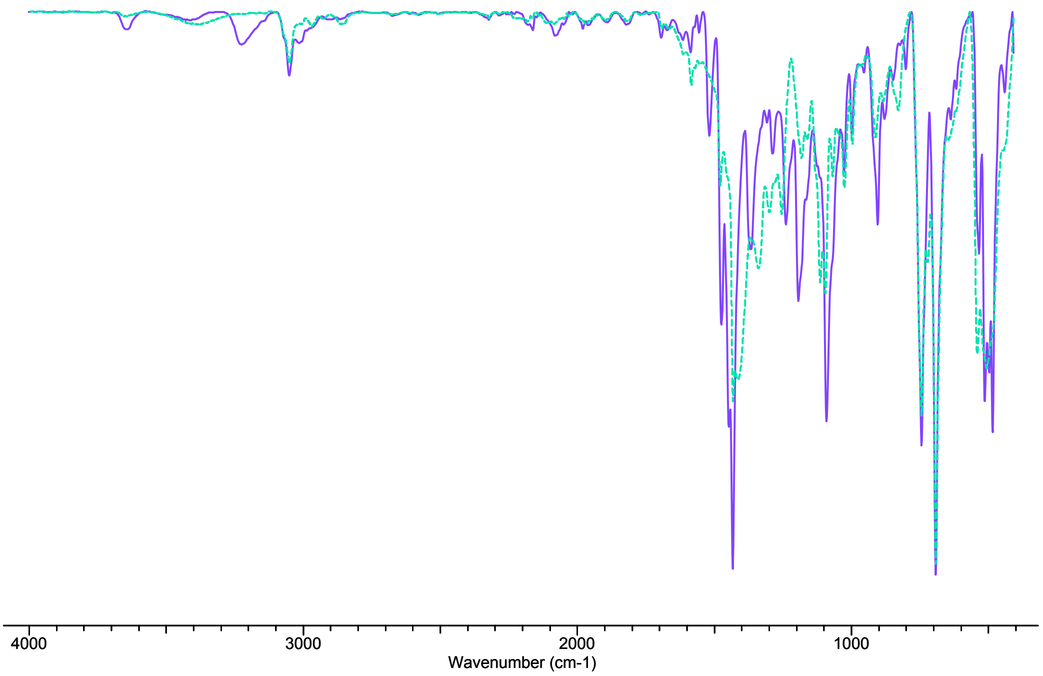

Figure S17. Superposition of the infrared spectra of $[Cu_4L_2]$ complex obtained in bideprotonation conditions (dashed line, green) and $[Cu_2(HL)_2]$ **2** complex (solid line, violet).

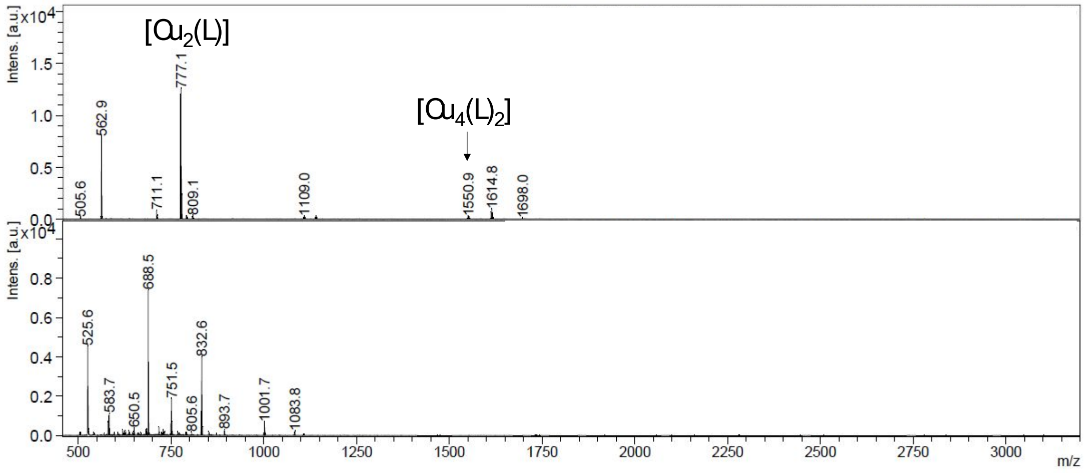

Figure S18. Mass spectrum of the tetranuclear copper complex, $[Cu_4(L)_2]$.

5. Cationic mesocates $[Cu_2(H_2L)_2](PF_6)_2$ 5, $[Cu_2(H_2L)_2](BF_4)_2$ 6, $[Ag_2(H_2L)_2](PF_6)_2$ 7, $[Ag_4(HL)_2](NO_3)_2$ 8, and $[Au_2(H_2L)_2]Cl_2$ 9

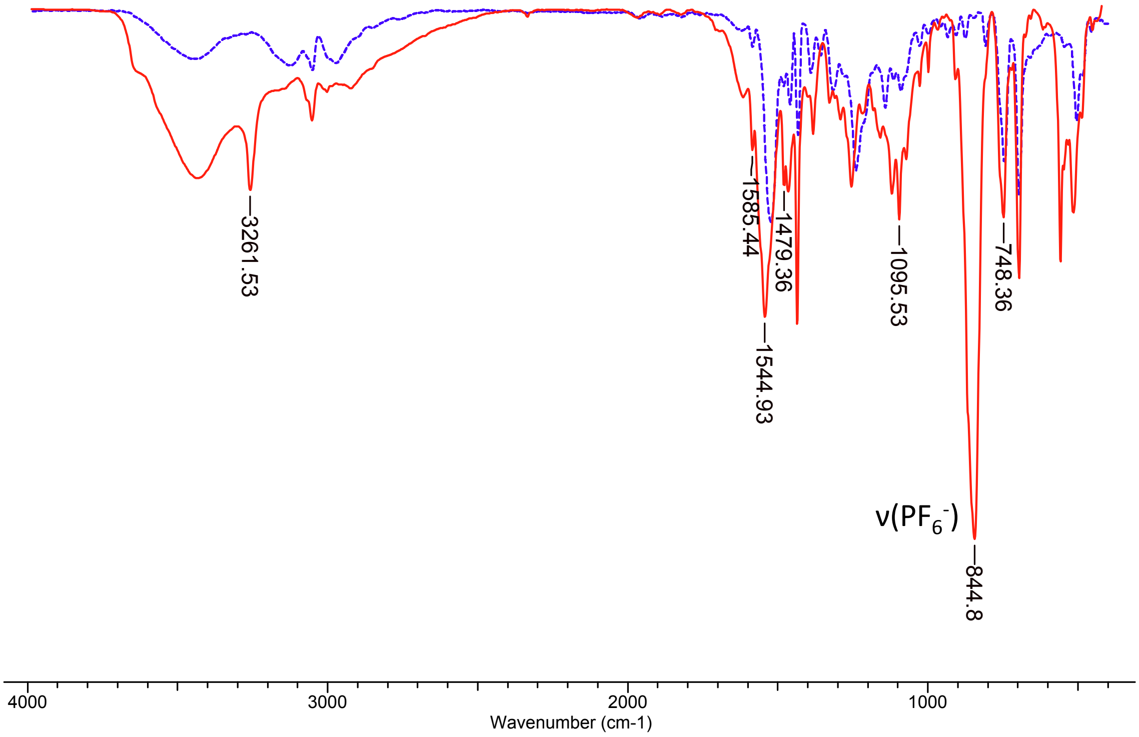

Figure S19. Superposition of the infrared spectra of the H_2L ligand **1** (dashed line, blue) and the $[Cu_2(H_2L)_2](PF_6)_2$ **5** mesocate (solid line, orange).

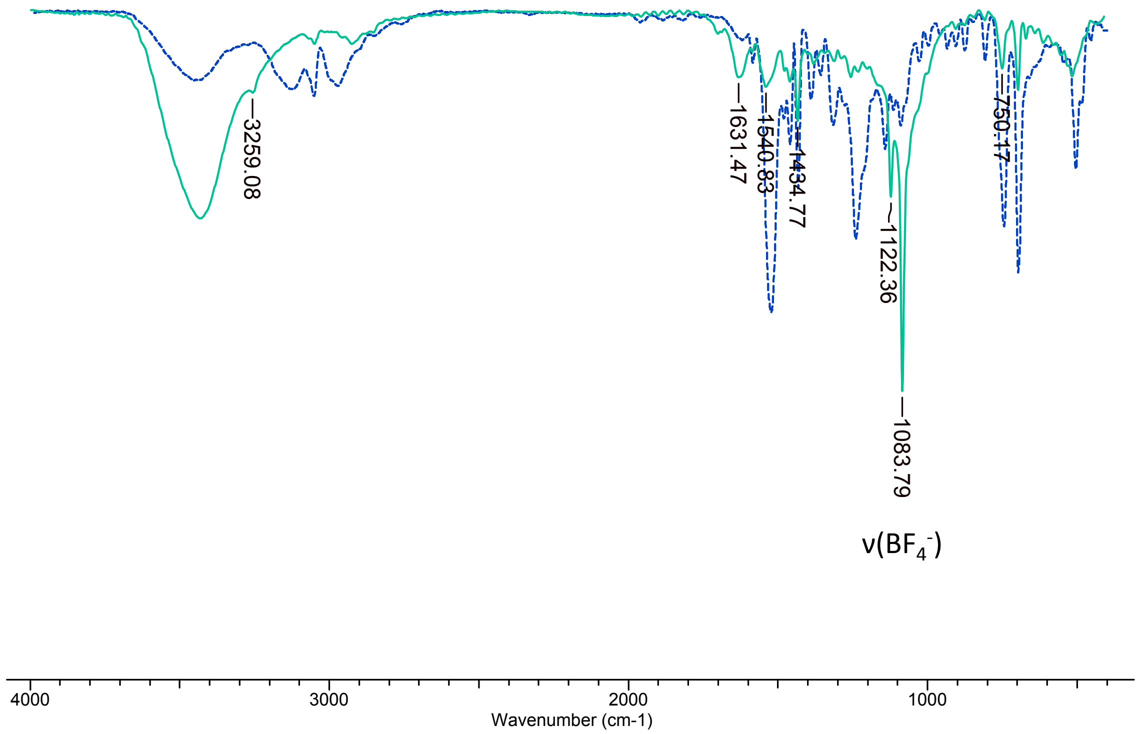

Figure S20. Superposition of the infrared spectra of the H_2L ligand **1** (dashed line, blue) and the $[Cu_2(H_2L)_2](BF_4)_2$ **6** mesocate (solid line, green).

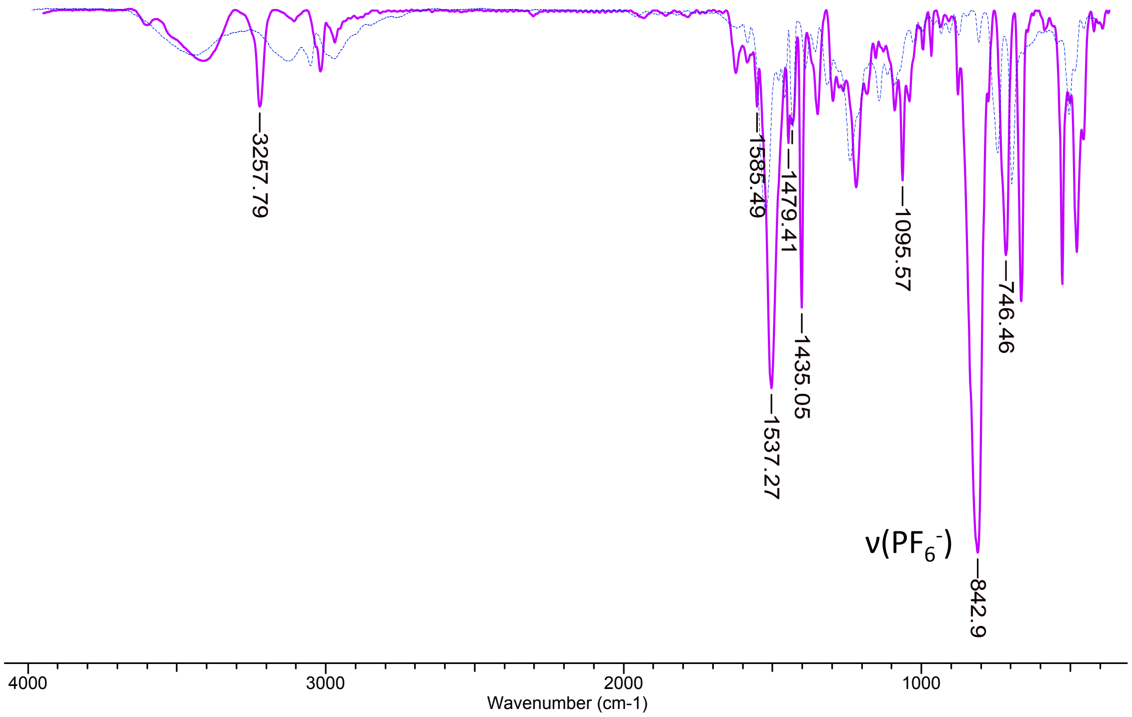

Figure S21. Superposition of the infrared spectra of the H_2L ligand **1** (dashed line, blue) and the $[Ag_2(H_2L)_2](PF_6)_2$ **7** mesocate (solid line, violet).

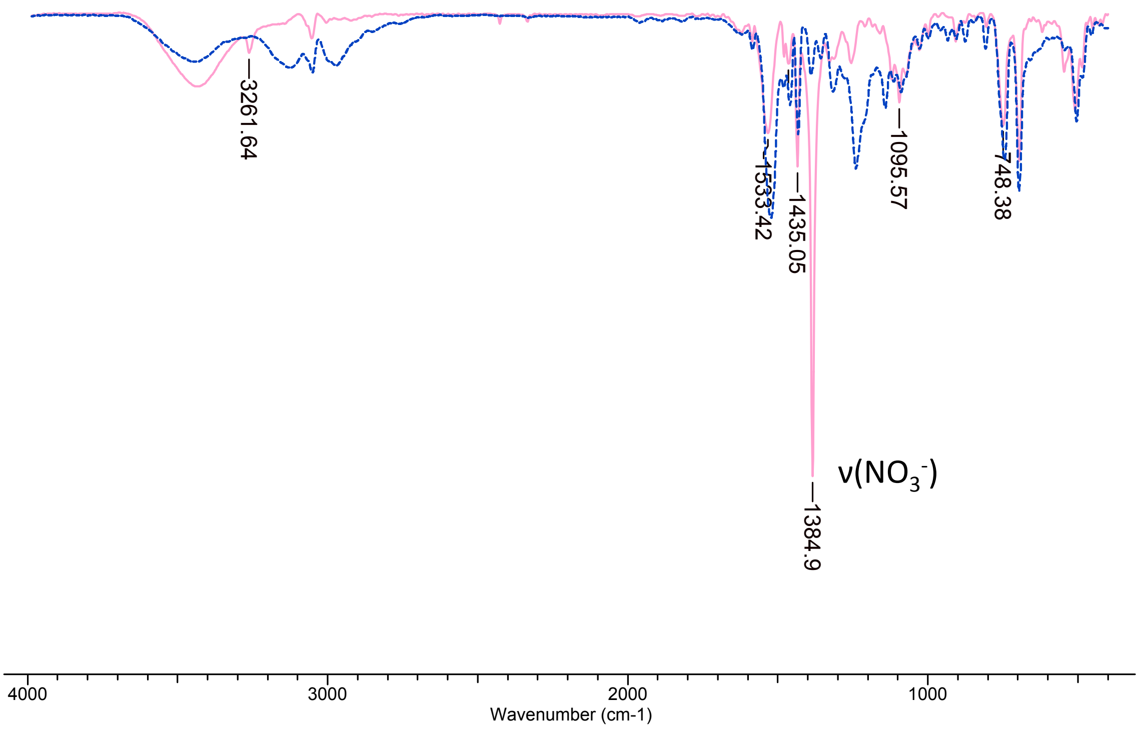

Figure S22. Superposition of the infrared spectra of the H_2L ligand **1** (dashed line, blue) and the $[Ag_4(HL)_2](NO_3)_2$ **8** mesocate (solid line, pink).

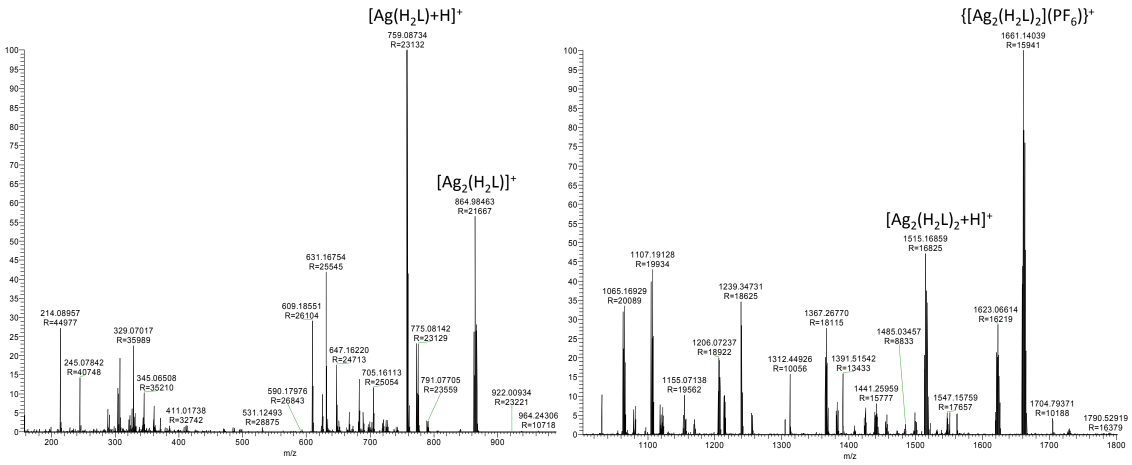

Figure S23. Mass spectrum of the $[Ag_2(H_2L)_2](PF_6)_2$ **7** mesocate.

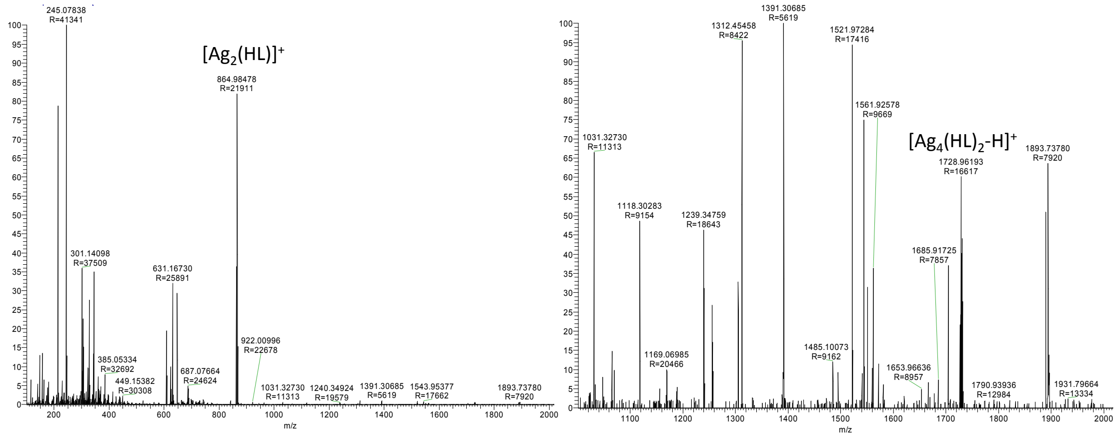

Figure S24. Mass spectrum of the $[Ag_4(HL)_2](NO_3)_2$ **8** mesocate.

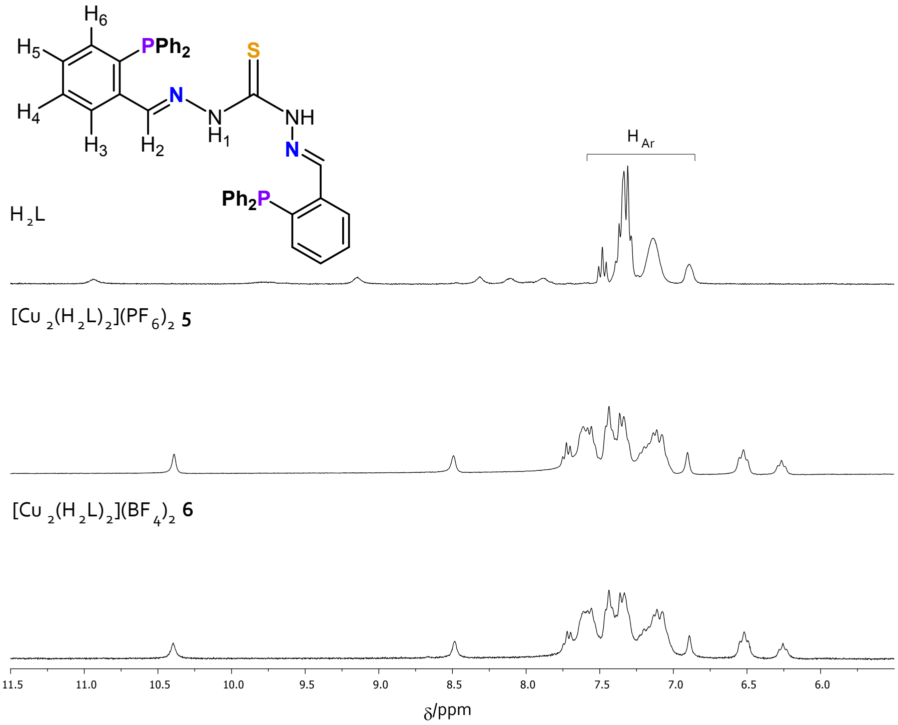

Figure S25. ^{1}H NMR spectrums of the copper(I) cationic mesocates **5** and **6** (CD_3CN-d$_3$, 298 K).

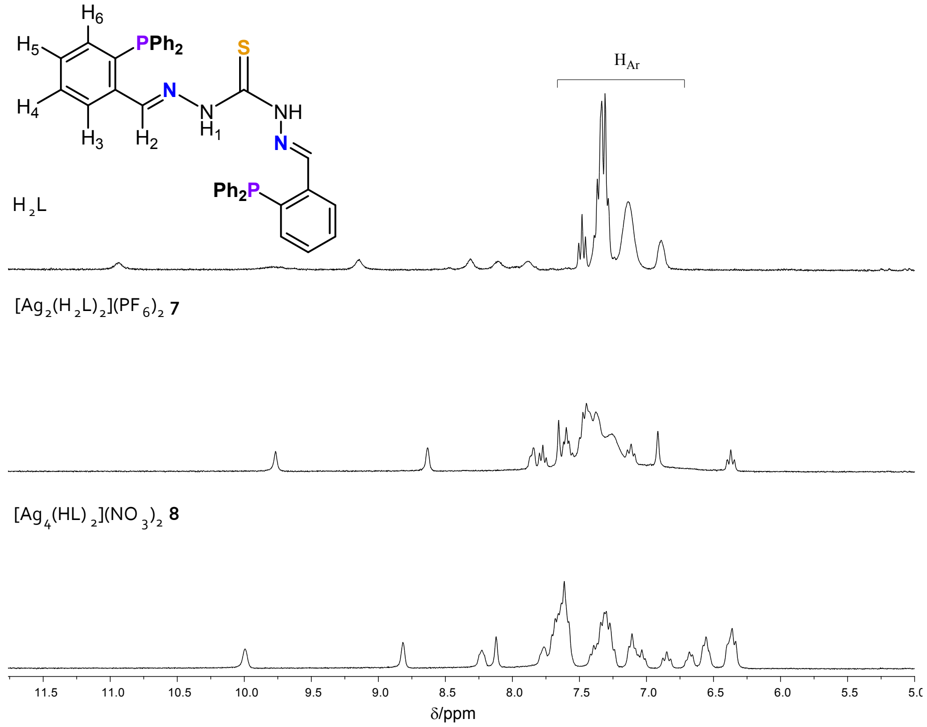

Figure S26. ^{1}H NMR spectrums of the silver(I) cationic mesocates **7** and **8** (CD_3CN-d_3, 298 K).

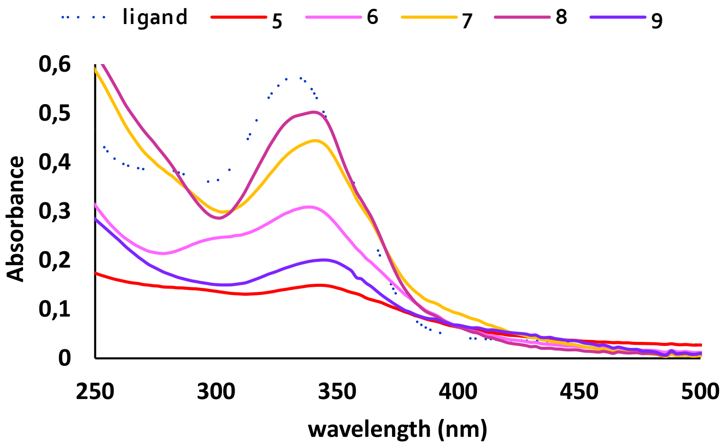

Figure S27. Superposition of the UV-Vis absorption spectra of the ligand and the ionic complexes 5-9 (CD_3CN-d_3, 298 K).

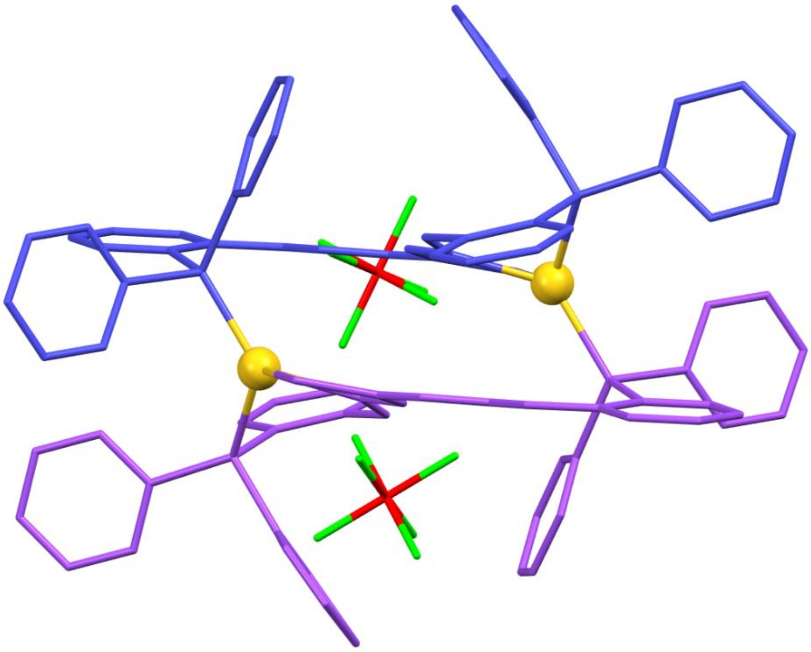

Figure S28. Sticks diagram of the dicationic copper(I) mesocate $[Cu_2(H_2L)_2](PF_6)_2{\cdot}7CH_3OH$ **5***.

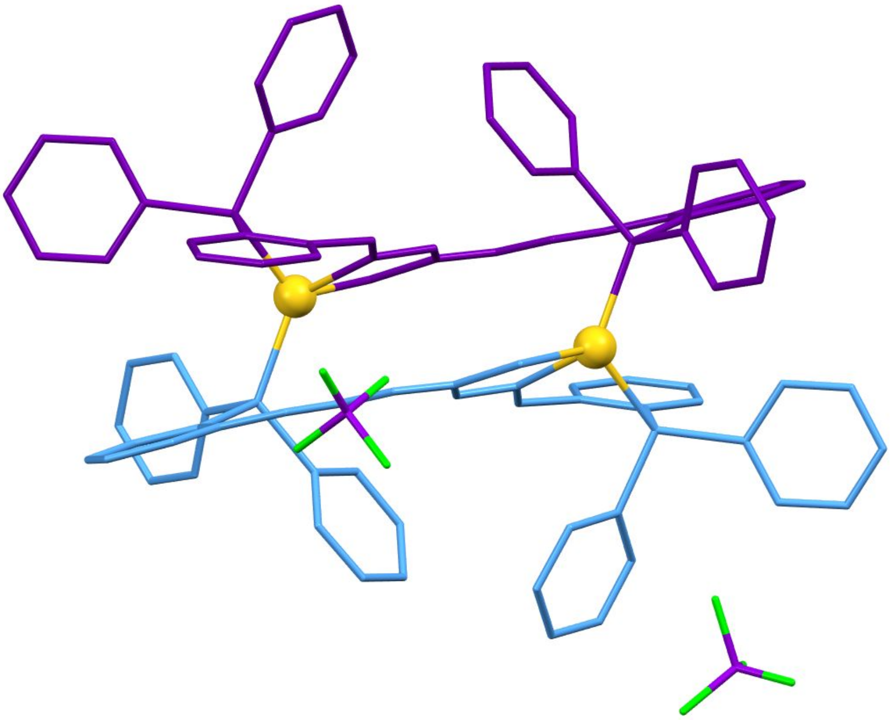

Figure S29. Sticks diagram of the dicationic copper(I) mesocate $[Cu_2(H_2L)_2](BF_4)_2{\cdot}5CH_3CN{\cdot}$**6***.

6. Crystallographic data of mesocates 2*–9*

Crystals **2***, **3*** and **5*–9*** were isolated from the mother liquors resulting from the synthesis of the corresponding solids. Crystal **4*** was obtained by recrystallization of the solid **4** in a mixture of chloroform/hexane.

Crystal data of compounds **2*–9*** were collected at 100(2) K on a Bruker Kappa APPEX II diffractometer with a CCD area detector, using graphite monochromated MoK(α) radiation (λ = 0.71073 Å). The data was collected and treated with APPEX2 (BRUKER AXS) software. In all cases, empirical absorption correction (SADABS)[1] was applied to the collected reflections. Structures **2***, **6*** and **7*** were solved by SIR2004,[2] **3*** was solved by DIRDIF2008,[3] **4*** was solved using SIR-92,[4] **5*** was solved by SIR-97[4] and **8*** and **9*** were solved by DIRDIF-99.[5] SHELXL program package[6] was used to refine by a full-matrix least-squares procedure based on F^2. All non-hydrogen atoms were located and refined anisotropically. The hydrogen atoms were included in the model at geometrically calculated positions and refined using a riding model. Molecular graphics include in the manuscript and the SI files were prepared with the MERCURY program.[7]

§ Crystallographic data for $[Cu_2(HL)_2]\cdot 3.5CH_3CN\cdot$**2***: $(2(C_{92}H_{82}N_{15}P_4S_2Cu_2))$, Mw = 3140.30 g/mol, crystal dimensions: 0.22 × 0.09 × 0.04 mm^3, triclinic, $P\bar{1}$, a = 12.0275(7), b = 16.3832(9), c = 19.7721(12) Å, α = 77.999(3), b = 84.996(4), γ = 85.670(4) º, V = 3789.8(4) Å^3, Z = 1, m = 0.76 mm^{-1}, Radiation l(Mo-K$_a$) = 0.7107 Å, T = 100 K, reflections measured/uniques 46596/10884 (R_{int} = 0.122), R = 0.054, wR = 0.115, GOF = 1.006, max/min residual density 0.45/-0.53 e.Å^{-3}. CCDC number 2145597.

§§ Crystallographic data for $[Ag_2(HL)_2]\cdot 4CH_3CN\cdot$**3***: $(C_{86}H_{74}N_{12}P_4S_2Ag_2)$, Mw = 1679.31 g/mol, crystal dimensions: 0.16 × 0.12 × 0.08 mm^3, Orthorhombic, $Pbca$, a = 19.633(2), b = 14.3471(17), c = 28.168(3) Å, α = 90, b = 90, γ = 90 º, V = 7934.4(16) Å^3, Z = 4, m =

0.68 mm^{-1}, Radiation l(Mo-K$_a$) = 0.7107 Å, T = 100 K, reflections measured/uniques 55045/7248 (R_{int} = 0.135), R = 0.054, wR = 0.141, GOF = 1.006, max/min residual density 1.23/-1.00 e.Å^{-3}. CCDC number 2145595.

§§§ Crystallographic data for [$Au_2(HL)_2$]·8$CHCl_3$·C_6H_{14} **4***: ($C_{86}H_{74}N_8P_4S_2Au_2Cl_{24}$), Mw = 2734.4 g/mol, crystal dimensions: 0.4 × 0.1 × 0.04 mm^3, Monoclinic, $C2/c$, a = 20.0394(10), b = 17.0357(8), c = 31.4759(14) Å, α = 90, b = 91.153(3), γ = 90 º, V = 10743.2(9) Å^3, Z = 4, μ = 3.47 mm^{-1}, Radiation l(Mo-K$_a$) = 0.7107 Å, T = 100 K, reflections measured/uniques 85489/13341 (R_{int} = 0.069), R = 0.048, wR = 0.120, GOF = 1.05, max/min residual density 1.98/-1.76 e.Å^{-3}. CCDC number 2145596.

§§§§ Crystallographic data for [$Cu_2(H_2L)_2$](PF_6)$_2$·CH_3CN·2H_2O·**5***: ($C_{80}H_{71}N_9P_6S_2O_2F_{12}Cu_2$), Mw = 1795.52 g/mol, crystal dimensions: 0.14 × 0.13 × 0.09 mm^3, Triclinic, $P\bar{1}$, a = 13.0530(4), b = 13.2574(4), c = 13.5878(4) Å, α = 113.673(2), β = 98.101(2), γ = 99.101(2) º, V = 2071.34(11) Å^3, Z = 1, μ = 0.758 mm^{-1}, Radiation λ(Mo-K$_a$) = 0.7107 Å, T = 100 K, reflections measured/uniques 38739/10287 (R_{int} = 0.0384), R = 0.062, wR = 0.0821, GOF = 1.018, max/min residual density 0.565/-0.368 e.Å^{-3}. CCDC number 2179776.

§§§§§ Crystallographic data for [$Cu_2(H_2L)_2$](BF_4)$_2$·5CH_3CN·**6***: ($C_{88}H_{79}N_{13}P_4S_2B_2F_8Cu_2$), Mw = 1795.52 g/mol, crystal dimensions: 0.11 × 0.11 × 0.09 mm^3, Monoclinic, $P2_1$, a = 15.9044(7), b = 14.4194(5), c = 19.5499(9) Å, α = 90, β = 109.402(2), γ = 90 º, V = 4228.8(3) Å^3, Z = 2, μ = 0.7 mm^{-1}, Radiation λ(Mo-K$_a$) = 0.7107 Å, T = 100 K, reflections measured/uniques 61387/10080 (R_{int} = 0.0406), R = 0.067, wR = 0.0867, GOF = 1.025, max/min residual density 0.584/-0.544 e.Å^{-3}. CCDC number 2181449.

§§§§§§ Crystallographic data for [$Ag_2(H_2L)_2$](PF_6)$_2$·6CH_3CN·**7***: ($C_{90}H_{82}N_{14}P_6S_2F_{12}Ag_2$), Mw = 2053.38 g/mol, crystal dimensions: 0.14 × 0.11 × 0.03 mm^3, Monoclinic, $P2_1$, a =

11.5189(5), *b* = 14.3109(6), *c* = 27.8690(10) Å, *α* = 90, *β* = 100.086(2), *γ* = 90 º, *V* = 4523.1(3) Å^3, *Z* = 2, *μ* = 0.664 mm^{-1}, Radiation *λ*(Mo-K$_a$) = 0.7107 Å, *T* = 100 K, reflections measured/uniques 70419/9248 (R_{int} = 0.0467), *R* = 0.0898, *wR* = 0.0877, *GOF* = 1.015, max/min residual density 0.556/-0.546 e.Å^{-3}. CCDC number 2181440.

§§§§§§§ Crystallographic data for $[Ag_4(HL)_2](NO_3)_2 \cdot 4CH_3OH \cdot$ **8***: ($C_{82}H_{78}N_{10}P_4S_2O_6Ag_4$), *Mw* = 1983.02 g/mol, crystal dimensions: 0.15 × 0.11 × 0.03 mm^3, Monoclinic, *P*2$_1$, *a* = 14.2322(6), *b* = 16.2322(5), *c* = 17.5170(7) Å, *α* = 90, *β* = 97.118(2), *γ* = 90 º, *V* = 4015.6(3) Å^3, *Z* = 2, *μ* = 1.16 mm^{-1}, Radiation *λ*(Mo-K$_a$) = 0.7107 Å, *T* = 100 K, reflections measured/uniques 75345/22800 (R_{int} = 0.0746), *R* (all data) = 0.0821, *wR* (all data) = 0.0828, *GOF* = 0.994, max/min residual density 0.739/-0.888 e.Å^{-3}. CCDC number 2181452.

§§§§§§§§ Crystallographic data for $[Au_2(H_2L)_2]Cl_2 \cdot 6.2CH_3OH$ **9***: ($C_{84.20}H_{88.80}N_8P_4S_2O_{6.20}Au_2Cl_2$), *Mw* = 1964.87 g/mol, crystal dimensions: 0.10 × 0.09 × 0.08 mm^3, Monoclinic, *P2/c$_1$*, *a* = 13.7470 (3), *b* = 25.6800 (5), *c* = 12.7772 (2) Å, *α* = 90, *β* = 112.5922 (9), *γ* = 90 º, *V* = 4164.51 (14) Å^3, *Z* = 2, *μ* = 3.767 mm^{-1}, Radiation *λ*(Mo-K$_a$) = 0.7107 Å, *T* = 100 K, reflections measured/uniques 93414/10338 (R_{int} = 0.069), *R* = 0.0521, *wR* = 0.0594, *GOF* = 1.035, max/min residual density 1.085/-1.17 e.Å^{-3}. CCDC number 2181125.

Bond distances (Å)			
**Cu1—N1**	2.075 (4)	**Cu1—P1**	2.2480 (16)
**Cu1—P4**	2.2398 (16)	**Cu1—S1**	2.3000 (15)
**Bond angles (°)**			
**N1—Cu1—P4**	122.26 (13)	**N1—Cu1—P1**	85.16 (12)
**P4—Cu1—P1**	123.44 (6)	**N1—Cu1—S1**	84.70 (12)
**P4—Cu1—S1**	116.86 (5)	**P1—Cu1—S1**	113.85 (6)

Table S2. Main bond distances (Å) and angles (°) in $[Cu_2(HL)_2]\cdot 3.5CH_3CN$ **2***.

Bond distances (Å)			
**Ag1—P2^{i}**	2.4268 (14)	**Ag1—P1**	2.4870 (15)
**Ag1—S1**	2.4975 (15)	**P2—Ag1^{i}**	2.4269 (14)
**Bond angles (°)**			
**P2^{i}—Ag1—P1**	121.21 (5)	**P2^{i}—Ag1—S1**	124.48 (5)
**P1—Ag1—S1**	112.48 (5)		

Table S3. Main bond distances (Å) and angles (°) in $[Ag_2(HL)_2]\cdot 4CH_3CN$ **3***.

Bond distances (Å)			
**Au1—P2^{i}**	2.2851 (15)	**Au1—P1**	2.3443 (13)
**Au1—S1**	2.5380(13)		
**Bond angles (°)**			
**P2i—Au1—P1**	134.51 (5)	**P2^{i}—Au1—S1**	119.06 (5)
**P1—Au1—S1**	106.42 (5)		

Table S4. Main bond distances (Å) and angles (°) in $[Au_2(HL)_2]\cdot 8CHCl_3\cdot C_6H_{14}$ **4***.

Bond distances (Å)			
**Cu1–S1**	2.3061 (6)	**Cu1–N1**	2.1674 (17)
**P1–Cu1**	2.2570 (6)	**Cu1–P2**	2.2449 (6)
**Bond angles (°)**			
**P2–Cu1–P1**	119.02 (2)	**N1–Cu1–P2**	133.17 (5)
**N1–Cu1–P1**	82.81 (5)	**N1–Cu1–S1**	83.78 (5)
**P2–Cu1–S1**	109.73 (2)		

Table S5. Main bond distances (Å) and angles (°) in $[Cu_2(H_2L)_2](BF_4)_2 \cdot CH_3CN \cdot 2H_2O$ **5***.

Bond distances (Å)			
**Cu1–S1**	2.3110 (6)	**Cu1–N1**	2.1430 17
**P1–Cu1**	2.2553 (6)	**Cu1–P2**	2.2479 6
**Bond angles (°)**			
**P2–Cu1–P1**	120.94 (2)	**N1–Cu1–P2**	127.16 (5)
**N1–Cu1–P1**	83.70 (5)	**N1–Cu1–S1**	84.21 (5)
**P2–Cu1–S1**	109.93 (2)		

Table S6. Main bond distances (Å) and angles (°) in $[Cu_2(H_2L)_2](BF_4)_2 \cdot 5CH_3CN \cdot$ **6***.

Bond distances (Å)			
**Ag1–S1**	2.5213 (11)	**Ag1–P1**	2.4669 (10)
**Ag1–P2^i**	2.4236 (10)		
**Bond angles (°)**			
**P2i—Ag1—P1**	134.51 (5)	**P2^i—Ag1—S1**	119.06 (5)
**P1—Ag1—S1**	106.42 (5)		

Table S7. Main bond distances (Å) and angles (°) in $[Ag_2(H_2L)_2](PF_6)_2 \cdot 6CH_3CN \cdot$ **7***.

Bond distances (Å)			
**Ag1–P1**	2.3968 (14)	**Ag1–N1**	2.519 (4)
**Ag1–N3**	2.413 (4)	**Ag1–S2**	2.4762 (14)
**Ag1-Ag2**	3.3249 (6)	**Ag2–P2**	2.3918 (13)
**Ag2–N8**	2.411 (4)	**Ag2–S2**	2.4828 (13)
**Ag3–P3**	2.4065 (14)	**Ag3–N5**	2.566 (4)
**Ag3–N7**	2.533 (4)	**Ag3–S1**	2.4690 (14)
**Bond angles (°)**			
**N3—Ag1—S2**	104.87 (10)	**P1—Ag1—N1**	77.23 (10)
**N3—Ag1—N1**	64.96 (14)	**P2—Ag2—N8**	126.04 (11)
**P2—Ag2—S2**	148.91 (5)	**P3—Ag3—S1**	140.18 (5)
**P3—Ag3—N7**	114.67 (10)	**S1—Ag3—N7**	103.38 (10)
**N4—Ag4—P4**	129.72 (10)	**N4—Ag4—S1**	76.38 (10)
**P4—Ag4—S1**	150.19 (5)		

Table S8. Main bond distances (Å) and angles (°) in $[Ag_4(HL)_2](NO_3)_2\cdot4CH_3OH\cdot$**8***.

Bond distances (Å)			
**P1—Au1**	2.3508 (9)	**P2—Au1**	2.2961 (9)
**S1—Au1**	2.5741 (9)		
**Bond angles (°)**			
**$P2^i$—Au1—P1**	133.46 (3)	**$P2^i$—Au1—S1**	118.45 (3)
**P1—Au1—S1**	107.95 (3)		

Table S9. Main bond distances (Å) and angles (°) in $[Au_2(H_2L)_2]Cl_2\cdot6.2CH_3OH$ **9***.

7. References

(1) Sheldrick, G. M. *Program for Scaling and Correction of Area Detector Data*, University.; 1996.

(2) Burla, M. C.; Caliandro, R.; Camalli, M.; Carrozzini, B.; Cascarano, G. L.; De Caro, L.; Giacovazzo, C.; Polidori, G.; Spagna, R. SIR2004: An Improved Tool for Crystal Structure Determination and Refinement. *J. Appl. Crystallogr.* **2005**, *38* (2), 381–388. https://doi.org/10.1107/S002188980403225X.

(3) Beurskens, P. T.; Beurskens, G.; de Gelder, R.; Garcia-Granda, S.; Gould, R. O.; Smits, J. M. *The DIRDIF2008 Program System, Crystallography Laboratory*, University.; 2008.

(4) Altomare, A.; Burla, M. C.; Camalli, M.; Cascarano, G. L.; Giacovazzo, C.; Guagliardi, A.; Moliterni, A. G. G.; Polidori, G.; Spagna, R. SIR97: A New Tool for Crystal Structure Determination and Refinement. *J. Appl. Crystallogr.* **1999**, *32* (1), 115–119. https://doi.org/10.1107/S0021889898007717.

(5) Beurskens, P. T.; Admiraal, G.; Beurskens, G.; Bosman, W. P.; de Gelder, R.; Israel, R.; Smits, J. M. M. *The DIRDIF-99 Program System, Technical Report of the Crystallography Laboratory*, University.; 1999.

(6) Sheldrick, G. M. A Short History of SHELX. *Acta Crystallogr. Sect. A Found. Crystallogr.* **2008**, *64* (1), 112–122. https://doi.org/10.1107/S0108767307043930.

(7) Macrae, C. F.; Bruno, I. J.; Chisholm, J. A.; Edgington, P. R.; Mccabe, P.; Pidcock, E.; Rodriguez-monge, L.; Taylor, R.; Streek, J. Van De; Wood, P. A. Mercury CSD 2 . 0 – New Features for the Visualization and Investigation of Crystal Structures. *J. Appl. Crystallogr.* **2008**, 466–470. https://doi.org/10.1107/S0021889807067908.
